# Exposure and response of satellite-tagged Blainville’s beaked whales to mid-frequency active sonar off Kaua‘i, Hawai‘i

**DOI:** 10.1186/s40462-025-00550-9

**Published:** 2025-04-21

**Authors:** E. Elizabeth Henderson, Michaela A. Kratofil, Robin W. Baird, Cameron R. Martin, Annette E. Harnish, Gabriela C. Alongi, Steve W. Martin, Brandon L. Southall

**Affiliations:** 1 NIWC Pacific, San Diego, CA USA; 2 Cascadia Research Collective, Olympia, WA USA; 3 National Marine Mammal Foundation, San Diego, CA USA; 4 Southall Environmental Associates, Inc.,, Santa Cruz, CA USA

**Keywords:** Blainville’s beaked whales, Satellite tags, Mid-frequency active sonar, Behavioral response, Dive behavior, Movement behavior

## Abstract

**Background:**

Beaked whale response to Navy sonars is a global concern due to past stranding events coinciding with training activity. Often, controlled exposure experiments involve tagging cetaceans with short-term, high-resolution tags and exposing them to relatively short, single bouts of mid-frequency active sonar (MFAS). In contrast, longer-duration satellite-transmitting tags deployed around Navy ranges enables behavioral response studies of animals exposed to realistic Navy training activities over extended periods and spatial scales, with multiple exposures to different sources.

**Methods:**

To study their behavior relative to extended periods of realistic Navy training, satellite-transmitting tags were deployed on four Blainville’s beaked whales (*Mesoplodon densirostris*) on the Pacific Missile Range Facility (PMRF) off Kaua‘i. Tags were deployed in 3 years, ahead of Submarine Command Courses (SCCs) with multiple sources of MFAS. Dive behavior of two tagged together were compared to acoustically detected group vocal periods (GVPs) on the range. Pre-exposure dive behavior metrics were compared to those during exposures. Horizontal movement behavior metrics were analyzed using Kruskal–Wallis non-parametric and Tukey–Kramer multiple comparison tests.

**Results:**

Two whales remained together and highly synchronized in their dive and movement behavior until the onset of MFAS, at which time they appeared to separate. Twenty-three deep foraging dives were matched to GVPs, including three during MFAS. Of the dive behavior metrics, only the depth of one intermediate dive during an exposure was outside the 95th percentile of baseline behavior. Three of the four movement behavior metrics (75%) were atypical relative to baseline for at least one whale across SCC phases, but response varied by individual. However, throughout the SCCs, the whales remained within tens of kilometers of PMRF, near areas used before and after SCCs.

**Conclusions:**

These data demonstrate some apparent short-term changes to dive behavior and horizontal movement in response to MFAS. However, these beaked whales did not demonstrate sustained avoidance responses, remaining in the area west of the range during MFAS and in two cases returning to the range after the SCC. Additional tagging and photo-identification studies are critical to understand Blainville’s beaked whale habitat use and residency and to assess the potential impact of repeated exposures to MFAS.

**Supplementary Information:**

The online version contains supplementary material available at 10.1186/s40462-025-00550-9.

## Multilingual abstract

Please see Supplemental Materials 2 for a translation of the abstract into Hawaiian.

## Background

Compared to chronic and acute noise exposures from global shipping traffic, seismic survey operations, and offshore alternative energy developments, sounds from US Navy training and testing activities generally occur in limited intervals and locations [1, 36]. An exception to this is on active US Navy ranges, where training and testing activities that may use mid-frequency active sonar (MFAS) occur with some regularity [1, 36]. Marine mammal monitoring on US Navy ranges creates unique circumstances to conduct opportunistic behavioral response studies on a variety of federally-protected species of interest (e.g., [25, 34, 35, 39, 40]). Monitoring also provides insight into resident populations to explore long-term demographics and potential impacts of repeated exposures to MFAS (e.g., [15, 19, 27, 29, 78]). While a variety of baleen and toothed whale species occupy these ranges, a species group of particular interest is the family of beaked whales (*Ziphidae*). Beaked whales have demonstrated an apparent behavioral sensitivity to MFAS, with a series of strandings in the late 1990s and early 2000s after several major multi-national navy training exercises [33, 17, 20, 26, 30, 31, 70]. These strandings may have resulted from the way beaked whales respond to sonar when conducting their deep foraging dives, coupled with specific oceanographic and bathymetric features [13, 20, 21, 41]. By tagging beaked whales that reside on or near Navy ranges concurrently with Navy training and testing activities, behavioral responses to MFAS in realistic training scenarios (e.g., involving multiple, overlapping transmission of different sources over days) can be observed and quantified.

Initial experimental studies of behavioral responses by beaked whales to MFAS (see [76]) were conducted using tags that can collect fine-scale movement and acoustic data such as DTAGs [48]. These studies have advantages in terms of experimental control over repeated, consistent exposure conditions, and high-resolution tags provide a level of detail that can’t be obtained through other methods. These studies also obtained the first empirical results of behavioral responses to MFAS. However, these studies were often conducted with simulated, projected MFAS-like sounds from experimental sources and so by necessity were often done in relatively close proximity (1–3 km) to the whales to achieve received levels (RLs) comparable to hull-mounted MFAS (e.g. [22]). Two Blainville’s beaked whales were tagged at the Atlantic Undersea Test and Evaluation Center (AUTEC) in the Bahamas, and exposed to simulated MFAS, a pseudo-random noise signal in the same bandwidth, and playbacks of killer whale (*Orcinus* spp) calls to determine what features of the signals might lead to a response (e.g., any signal in that bandwidth or with other characteristics of predator calls such as frequency modulation; [76]). The whales responded to MFAS at 138 dB re 1 μPa (all levels reported herein as root-mean-square), pseudo-random noise at 142 dB re 1 μPa, and killer whale calls at 98 dB re 1 μPa. Similarly, two goose-beaked whales (*Ziphius densirostris*) tagged off southern California responded to simulated MFAS at 98 and 127 dB re 1 μPa but did not respond to more distant surface ship hull-mounted MFAS at comparable RLs (78–106 dB re 1 μPa from a distance of 118 km; [22]), while a Baird’s beaked whale (*Berardius bairdii*) tagged in the same study responded at 127 dB re 1 μPa at a distance of 2.7 km [73]. All three species of beaked whales responded with a cessation of foraging, indicated by a stop in echolocation clicks earlier than expected, movement away from the source location, and ascents from extended deep dives that were longer and slower than in normal dives. These results were the first experimental validation that beaked whales may be sensitive to MFAS, which may be due to a perceived similarity between MFAS and killer whale vocalizations [76]. These results also indicated that there may be a spatial relationship to a response as well, such that beaked whales may be able to discern the distance of the source and respond differently to more distant MFAS.

Additional opportunistic studies of beaked whale responses to MFAS have been conducted on multiple Navy ranges, and generally support the idea that there is a relationship between proximity to MFAS sources and the degree of behavioral response that animals display, with both distance to source and RL used as metrics to quantify this relationship. Some of these studies have focused solely on the use of Passive Acoustic Monitoring (PAM) to assess behavioral responses in the form of cessation of foraging. These data have been used in the development of behavioral response functions to estimate the probability of a response based on RL using aggregated group vocal period (GVPs, acoustic detections on one or more hydrophones of a presumed foraging group of beaked whales) data from one or more multi-day Submarine Command Course (SCC) [44, 66]. Another approach with PAM has been to quantify the number of GVPs detected on the range before, during, and after SCCs; consistent reductions in GVPs have been found during training activity both with and without the use of MFAS [39, 55, 56, 60, 76], although it cannot be determined through PAM if the whales are leaving the range or going silent.

Other range-based studies have used satellite-transmitting tags (hereafter “satellite tags”) to observe dive and movement behavior during training activity. Joyce et al. [49] examined the movement patterns of eight Blainville’s beaked whales that were satellite-tagged prior to a Navy training event on the Atlantic Undersea Testing and Evaluation Center (AUTEC) range in the Bahamas. Three of the animals were on the range when MFAS started; all three plus a fourth animal near the range moved 28–68 km away from the range at estimated RLs of 145 to 172 dB re 1 μPa but returned to the range within 1.7–3.9 days. Two of these tags also recorded dive data; both animals continued to conduct deep dives during the period of MFAS exposure, but the one that was tagged closer to the range did not dive as deep as they had before the MFAS and conducted more mid-depth dives [49]. Falcone et al. [28] combined data from 16 satellite-tagged goose-beaked whales on the Southern California Offshore Acoustic Range (SOAR) to assess impacts of both high-powered, hull-mounted MFAS as well as lower power helicopter-dipping MFAS. Both deep and shallow dive durations, as well as inter-deep dive interval (IDDI) durations, increased with proximity to the helicopter-dipping MFAS. IDDIs were also longer in the presence of hull-mounted MFAS, as were shallow dives, but the response was not as strong as in the presence of helicopter-dipping MFAS, or in the presence of both sources combined [28].

The acoustic environment of both the Joyce et al. [49] and Falcone et al. [28] studies may have influenced how animals responded to MFAS. Both ranges are located in deep basins adjacent to islands, where water depths reach 1800–2000 m, but the deep waters are surrounded by ridges and seamounts. To move out of the ensonified basin, beaked whales must travel tens of kms and from deep into shallow waters, where oceanographic factors may cause surface ‘ducting’ with enhanced propagation resulting in relatively higher exposure levels near the surface [21]. In contrast, the seafloor around the Pacific Missile Range Facility (PMRF), located off the island of Kaua‘i in the Hawaiian archipelago, forms a steep slope off the pinnacle island in all directions, and waters reach 5400 m in depth within 6–15 km from shore and remain deep. This underwater environment leads to a divergent soundscape at PMRF, which may lead to different behavioral responses than have been observed at AUTEC or SOAR. Alternatively, beaked whale behavioral responses to sonar may be similar in all locations, regardless of the sound field, bathymetry, prey dynamics, or individual exposure histories. For example, if the reaction is an instinctive anti-predator response to a sound with similar properties to killer whale whistles (e.g., [3, 76, 79]), then the resulting behavior should remain the same.

While RLs and behavioral responses have been previously estimated for other odontocete species exposed to MFAS at PMRF [11, 12, 40], beaked whales have only been studied acoustically [38, 44, 55, 56]. This paper describes the movement and dive behavior of four Blainville’s beaked whales satellite-tagged prior to three biannual SCC training events conducted at PMRF. These events included the use of multiple MFAS sources, including active sonobuoys and helicopter-dipping sonar, analyzed for the first time at PMRF. Novel methods were used to estimate dive and movement behavioral responses to MFAS. In addition, dives from the tagged animals were linked with acoustically detected GVPs on the range for the first time, allowing for comparisons and correlations between the two data streams to fully understand the acoustic scene of Blainville’s beaked whale foraging dives at PMRF.

## Methods

### Field operations and tag analysis

Tagging was undertaken from a 7.3-m rigid-hulled inflatable boat (RHIB) during field operations conducted between Kaua’i and Ni’ihau in February 2014, August 2021, and August 2022. Field operations were timed to occur immediately prior to SCCs, to maximize the likelihood of data being obtained for periods before, during, and after the SCC. At PMRF, SCCs are broken into five phases: Before; Phase A, a period of training activity that does not typically include surface ship hull-mounted, sonobuoy, or helicopter-dipping MFAS or large surface ship activity; the Interphase between Phase A and B with no training activity, although vessels may still be present in the area; Phase B, a period of training activity that may include all three sources of MFAS and large surface ship activity; and After. For analysis purposes, tag durations in the Before and After periods were truncated to 3 days. During encounters, information was recorded on group size, start and end time, and locations (see [10]). Photographs of all individuals within the groups were taken for individual identification, determination of age (based on the degree of scarring and relative size), and sex (based on presence or absence of erupted teeth). Photos were then compared to a long-term photo-identification catalog [6, 64] to assess sighting history and the potential for repeat tagging of individuals.

Tags included both location-only (SPOT5) and depth-transmitting SPLASH10-F tags (Wildlife Computers, Redmond, WA) in the Low Impact Minimally Percutaneous Electronic Transmitter configuration [2]. Tags were deployed with a Dan-Inject pneumatic projector and were attached with two gas-sterilized 4.4-cm surgical grade titanium darts (see [9, 68]). Tags were programmed to transmit during the 14 or 16 h of the day with the greatest density of satellite overpasses, based on Argos pass predictions obtained through Collecte Localisation Satellites (https://www.cls.fr/en/). Transmitted dive data from the SPLASH10-F tags included behavior logs recording the start and end time of dive and surface periods, maximum dive depths, and dive shape (U-, square-, or V-shaped). All tags were programmed to record maximum dive depths and durations for dives greater than or equal to 50 m and lasting longer than 30 s to reduce gaps in the behavior logs [69]. Dive behavior data were examined prior to analyses to ensure that the tags operated as intended and experienced no malfunctions that could invalidate the data (e.g., [64]). Surface periods were considered any time when the animal did not dive below 50 m; note that animals could be anywhere in the water column down to 50 m during a surface period. Two shore-based Argos receivers (Wildlife Computers Motes), one on Ni’ihau and one on Kaua’i, were also used to increase data throughput when tagged animals were within range of the receivers [45].

Argos and GPS location data were processed building on methods detailed in Kratofil et al. [52]. Briefly, Argos locations were processed through the Douglas Argos Filter via Movebank [23, 51] to remove erroneous locations. GPS locations were restricted to those with residual values less than 35 and time errors less than 10 s [24] and then processed through a speed filter. Resultant Argos and GPS locations were combined, and subsequently fitted to a continuous-time correlated random walk (CTCRW) movement model via the *crawl* package in R [46, 47] to predict locations at a regular time step for further analyses. Beaked whale location data are often sparse and characterized by comparatively poor location accuracy due to their long diving and cryptic surface behavior. While *crawl* incorporates positional uncertainty (i.e., Argos-derived position-specific error ellipse measurements) into the movement model, the sparseness of beaked whale tracks may not appropriately align with the underlying movement process of the CTCRW. Therefore, we fit each track to two different movement models to determine the best fit model: (1) the CTCRW, which is the default model fit by *crawl*; and (2) a Brownian movement model (BM), which was fit by setting the beta parameter (velocity autocorrelation) to three (Devin Johnson pers. comm.) and depicts a less correlated movement process [42]. The Akaike Information Criteria (AIC) was used to determine which model was a better fit for each tag. Argos error ellipse measures were incorporated into both models in the same manner to account for positional uncertainty. We specified error ellipse measurements for GPS locations based on the number of satellites used to derive the GPS location as done in Henderson et al. [40]. Best fit models selected for each individual tag were then used to predict locations at a 5 min interval for received-level analyses following Henderson et al. [40]. Predicted locations also include an estimated standard error (meters) in both x and y directions (easting and northing), which was used to account for positional uncertainty in received-level analyses. Lastly, any locations on or near land were re-routed around land using the 300-m isobath (as in [52]) using the *pathroutr* package [54].

### MFAS detection, localization, and modeling received levels

Detection and localization of MFAS was conducted following Henderson et al. [40] and is summarized here. A custom computer-based recorder collected acoustic data from PMRF bottom-mounted range hydrophones at a 96 kHz sample rate with 16-bit samples. Detection of MFAS transmissions occurred in two distinct mid-frequency bands between 1 and 10 kHz. Due to security considerations, exact frequency bands cannot be provided; however, the lower frequency band detected surface ship hull mounted MFAS (i.e., AN/SQS-53C) and helicopter dipping MFAS (i.e., AN/AQS-22) while the higher frequency band detected active sonobuoys (i.e., AN/SSQ-62). Nominal source levels at 1 m distance for these three MFAS types are: hull-mounted sonar at 235 dB re 1 µPa, helicopter-dipping sonar at 217 dB re 1 µPa, and active sonobuoys at 201 dB re 1 µPa [30]. Model-based localizations of MFAS transmissions were performed under the same suite of C+ + algorithms for whale calls described in detail by Martin et al. [58] and Martin et al. [59]. For all sources, a sonar bout was considered a period with MFAS with breaks between emissions less than 30 min. A bout could be made up of a single source or could have signals from multiple sources.

The estimation of RLs on whales from MFAS transmissions utilized methods described in detail in Henderson et al. [40]. Propagation modeling was done with the Peregrine parabolic equation propagation model developed by Oasis Ltd [37], based on the range-dependent acoustic model (RAM) [16]. The predicted x and y positional error from interpolated whale track locations were used to define a 95% confidence interval error ellipse to represent location uncertainty around each modeled whale location. The error ellipse was sampled with radial slices taken systematically in azimuth; modeling was performed across the full depth from 0 to 5400 m, and the distance of the longest radial slice for an MFAS transmission was used for all radials from the same MFAS transmission. To estimate the probable three-dimensional location of the animal at the time of MFAS transmissions requires modeling the animal’s location in the depth dimension over the 95% CI error ellipse for each modeled whale location. Animal depth was derived from the satellite tag data (See Dive Analysis section for more details on how this was modeled). When the satellite tags provided depth information, the modeled animal depth was utilized that corresponded to the time an MFAS transmission was received at the animal position, along with a percent of depth to represent uncertainties in depth. When no depth data were available (e.g., when behavior logs had a gap, or for the two tags without dive behavior logs), received levels were estimated for two depth regimes: shallow (0–54 m) and the remainder of the water column down to the typical dive depth for Blainville’s beaked whales (> 54 m to 1125 m) [7].

Each surface ship hull-mounted MFAS localization was joined to ship positional data if the localization time and position were within one second and 400 m of the given ship position. During a 5 min interval (the period from one predicted track location to the next), one transmission from each ship transmitting MFAS and its azimuthal radials were selected for propagation modeling if it was closest in time (within ± 2.5 min) and distance to a whale position. While the number of MFAS transmissions from all sources in a 5 min bin is sensitive, a stoplight colorization of the median RL is provided in RL plots to indicate relative MFAS activity in each 5 min bin with green being low, yellow moderate, and red high (Supplemental Material 1).

Localized MFAS transmissions from sonobuoys and helicopter dipping sonar were automatically tracked with an adapted algorithm [50] that has been utilized to acoustically track whale calls at PMRF (e.g., [25, 35, 58, 59]). The automated tracking algorithm first filters localizations by quality indicator thresholds, then groups localizations by recursively examining time and distance between localizations. This method was assessed and validated using data from a previous SCC; see Martin et al. [58] for details on that analysis and the user-defined values used for tracking. For each sonobuoy and dipping sonar track, a single transmission with the minimum number of error ellipse radials and closest update in time and distance to a whale was selected for propagation modeling. Since sonobuoys and helicopter dipping sonar typically transmit for short periods of time and have minimal movement during active periods, the modeled transmission loss from a single, mid-point transmission reasonably represents the rest of the transmissions that composed a sonobuoy or dipping sonar track.

### Dive analysis

The SPLASH10-F tag deployed in 2022 malfunctioned, invalidating the behavior log, and the SPOT5 tag in 2014 did not include dive data, leaving only two tags with usable dive data (MdTag020 and MdTag021). Using these data, coupled with the predicted tracks in 5 min intervals, full dive cycles were modeled using a custom Matlab program as described in Henderson et al. [40]. SPLASH10-F tags report a minimum and maximum estimate for the dive depth and for the dive duration metrics; the means of these values were taken for all analyses. First, minimum and maximum bottom times were estimated based on Wildlife Computer’s definitions of U-, V-, and square-shaped dives. Then, ascent and descent times were estimated based on the remaining time in the dive divided by two, and ascent and descent rates were determined using the mean maximum depth divided by the estimated ascent and descent times. These values were bounded by the ascent and descent rates reported by Baird et al. [7] such that if the estimated rates were lower than the published minimum rate, or were higher than the published maximum rate, then the published minimum or maximum rate was used. These values (minimum and maximum bottom time, ascent and descent times, and ascent and descent rates) were estimated for all dives. Dive behavior logs and Mote transmission logs were also examined for records of surface and dive periods. If data were missing, these periods were noted as well, while times from the transmission logs were utilized as known surface times.

Dive durations were interpolated by 60 points per dive, leading to a timestamp approximately every 30 s for a deep (> 300 m) dive and approximately every 10 s for an intermediate (typically ≤ 300 m) dive. Dive depths were modeled using the estimated ascent and descent rates at each interpolated timestamp, and these were combined with the surface predicted track for a full record for each whale. At each timestamp (either at 5 min intervals when at the surface or at the finer interpolated intervals of the dives), it was also noted whether that timestamp occurred during a dive, was at the surface but interpolated, or was at the surface with either an Argos or GPS update or Mote uplink. Finally, it was noted whether that timestamp occurred during a period of missing behavioral log data. In that case, it was assumed the animal was in the top 50 m of the water if the interpolated timestamp occurred within 1 min of an Argos or GPS location or an attempted tag transmission.

The minimum, mean, median, and maximum durations of both intermediate (50–300 m) and deep dives (> 300 m), as well as surface periods during which shallow dives (< 50 m) may have occurred, were calculated for the two tags with complete dive behavior logs. Consecutive intermediate dives and surface periods were also combined to identify inter-deep dive intervals (IDDI), their durations, and the number of intermediate dives that occurred in each IDDI, each of which have been behavioral parameters in which changes have been identified for beaked whales in previous studies with MFAS (e.g., [22, 28]).

### Group vocal period analysis

For the periods of time the Blainville’s beaked whales were known to be over the PMRF range hydrophones, the locations of deep dives for MdTag020 and MdTag021 were compared to acoustically detected GVPs. Hydrophones with GVPs within 10 min and 6 km of the 95% CI error ellipse from each predicted modeled track position were identified, to be certain that if the animal were truly located anywhere within the error ellipse it would still be detected on the hydrophone (a distance assumed to be 6 km based on McCarthy et al. [60]). Next, the raw acoustic data from each hydrophone with detections from each of the selected GVPs were examined for echolocation pulse onset and cessation times. These times were compared to the tag dive start and end times. If the start and end times of the GVPs fell within the start and end times of the tag dive, then the GVP was assumed to belong to the tagged whales’ group. Using the modeled dive profiles, the start and end depths of the GVPs were estimated. While tag clocks do have some drift, they were reset before deployment and the GVPs occurred within the first few days of deployment, so clock drift should not impact estimated dive start and end times. However, it should be noted that dive times are triggered at 3 m depth, so the tag dive start and end times will be shortened by 2–4 s on either end.

### Statistical analyses

To assess changes in dive behavior during MFAS exposures, percentile values were calculated from baseline dives (before MFAS) for a variety of strategically selected dive parameters including deep dive depth, deep dive duration, IDDI duration, mean IDDI intermediate dive duration, mean IDDI intermediate dive depth, and maximum IDDI intermediate dive depth. These parameters were selected based on previous behavioral response studies using high-resolution sound and movement tags (e.g., [71]). The values for the Phase B exposure dive data variables were compared to pre-MFAS dive values to evaluate how atypical they were relative to baseline (known no-sonar) behavior in pre-MFAS dives. While not a formal inferential statistical test, this percentile assessment was intended to provide a sense of the relative likelihood of parameters observed during exposure being common or uncommon of individual-specific parameters. We considered exposure dive parameters to be relatively atypical and indicative of a possible response if MFAS-exposure dive metrics fell outside the central 95th percentile (i.e., below or above the 2.5th and 97.5th percentile values) of baseline values for each diving metric. A diel analysis of dive patterns across phases was also conducted (See Supplemental Material 1).

To assess changes in individual spatial (horizontal) movement behavior, bearing (degrees), turning angle (radians), step length (distance between track locations in meters), and travel speeds (m/s) were calculated for each step along the predicted tracks using the *prep_data* function in the R package *bayesmove* [18]. To more comprehensively account for positional error, this analysis was conducted on 30 imputations of both the full predicted track record of all four animals and on the same 30 imputed tracks after removing intervals with gaps between updated positional data longer than 1 h. Multiple imputation of the tracks was completed in the *crawl* package; imputed tracks were generated from the movement parameters estimated from the best fit model (i.e., the same models fit to the data in the tagging methods above; [47]). The use of 30 imputations more comprehensively accounts for the spatial error of the predicted positions [61, 75], and the comparison of full tracks to tracks with only 1 h or less between position updates removes the potential for artificial smoothing between more temporally separated positions. The movement variables were then compared across the SCC phases defined previously for each whale. As these variables had non-normal distributions, Kruskal–Wallis tests were performed to compare the values across the five SCC phases, and Tukey–Kramer multiple comparison tests were performed to determine which periods were statistically different from each other. The results of the statistical tests were compared across the two track types (full and positional gaps ≤ 1 h) to determine if any of the movement variables had statistically significant changes across SCC phases with the longer location gaps removed, which should be an indication that the patterns in behavioral changes are real and not an artifact of smoothing the track between more distant positional updates.

## Results

### Tagging and baseline data

Two Blainville’s beaked whales were tagged in a group of five individuals in 2014, but data were only obtained from one of the two tags (MdTag017), deployed on an adult male. Of the five individuals photo-identified in the group, none had been previously documented or have been photographed subsequently. Two individuals were tagged in a group of seven individuals in 2021, one adult female (MdTag020) and one adult male (MdTag021). An adult female (MdTag022) in a group of seven individuals was tagged in 2022. Based on photo-identification, none of the individuals were tagged on more than one occasion. The adult male tagged in 2021 had previously been photo-identified off Kaua’i on two occasions, in March and June 2019 (Cascadia Research Collective, unpublished data). Note that this adult male was also photographed in 2024 and was observed in a group with another known female (Baird, unpublished data). There were two individuals in common between the groups tagged in 2021 and 2022 (MdTag022 and a juvenile, presumably her offspring). Of the combined 17 individuals photo-identified in the three encounters, none have been documented off other islands. Location data were obtained for 8.2 (MdTag017), 13.3 (MdTag020), 9.0 (MdTag021), and 24.3 (MdTag022) days. All times are presented in Hawaiian Standard Time (HST).

**Fig. 1:**
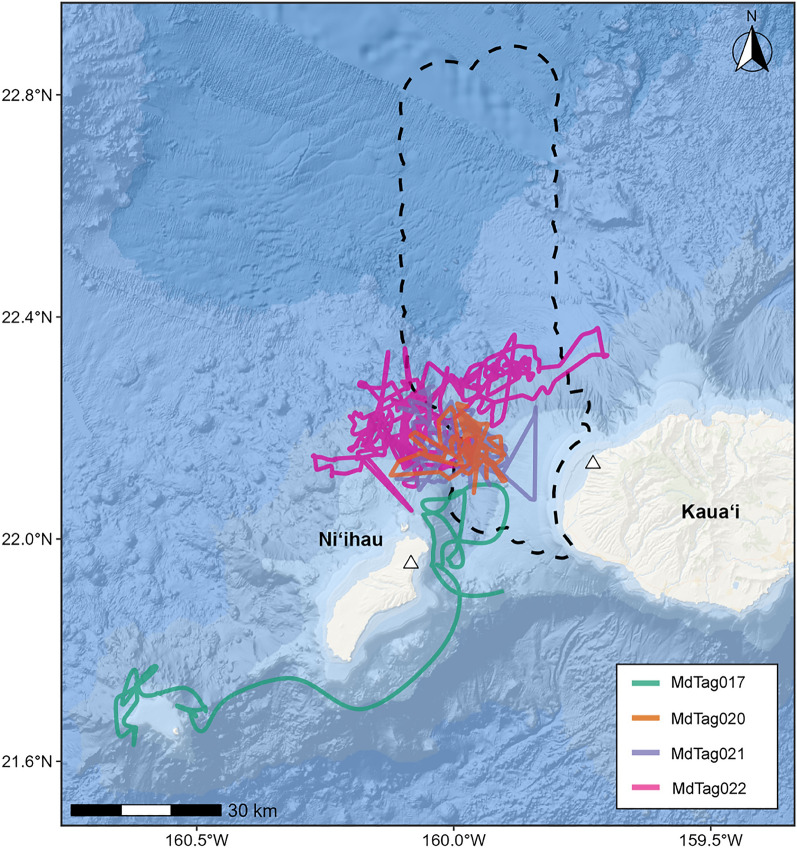
Tracks of tagged Blainville’s beaked whales (2014, green, *n* = 1; 2021, purple and orange, *n* = 2; 2022, pink, *n* = 1), with the outline of the PMRF range shown as a black dashed line for reference. The approximate locations of the Mote antennas are given as blue triangles

The Blainville’s beaked whale MdTag017 was tagged at PMRF on 4 Feb 2014 at 12:42; this tag transmitted until 12 Feb at 17:19. This individual remained within 5 km of the range for 3.5 days, spending time in the Kaulakahi Channel between Kaua‘i and Ni‘ihau, and only overlapped with the range hydrophones for 10 h on 7 February before heading southwest away from the range along Ni‘ihau and west to Ka‘ula Island (Fig. 1). This animal left the area before the start of any training and so did not have any MFAS exposures (Table 1).

**Table 1:** SCC phase times and passive acoustic monitoring data durations (in HST)

Phase	Start date	End date	Duration (hrs)
Before	11 Feb 2014 15:30	12 Feb 2014 18:00	26.5
Phase A	12 Feb 2014 18:00	14 Feb 2014 21:00	51.0
Interphase	14 Feb 2014 21:00	17 Feb 2014 19:00	70.0
Phase B	17 Feb 2014 19:00	20 Feb 2014 17:30	70.5
After	20 Feb 2014 17:30	20 Feb 2014 22:30	6.0
Before	8 Aug 2021 18:00	11 Aug 2021 17:59	72.0
Phase A	11 Aug 2021 18:00	13 Aug 2021 10:20	40.3
Interphase*	13 Aug 2021 10:21	17 Aug 2021 4:59	90.6
Phase B	17 Aug 2021 5:00	19 Aug 2021 14:30	57.5
After	19 Aug 2021 14:31	22 Aug 2021 14:30	72.0
Before	13 Aug 2022 16:00	16 Aug 2022 16:00	72.0
SCC A/B (mixed)	16 Aug 2022 6:30	20 Aug 2022 23:31	89.0
Interphase	20 Aug 2022 23:32	23 Aug 2022 12:50	73.3
SCC B/A (mixed)	23 Aug 2022 12:51	24 Aug 2022 15:53	27.0
After	24 Aug 2022 15:54	27 Aug 2022 15:54	72.0

Note that only 3 days before and after the SCCs in 2021 and 2022 are given to correspond with the Before and After periods used in the behavioral response analyses. *The Interphase in 2021 included a unit-level training activity on 8/15 that did not include MFAS

In 2021, two Blainville’s beaked whales were tagged in the same group on 11 Aug, the first (MdTag020) at 12:03 and the second (MdTag021) at 12:33; these animals were tagged a half day before the start of the SCC (Table 1). Phase A of the 2021 SCC was comprised of training activity but there were no active MFAS sources present. There was a 4-day period between the two phases of training activity (Interphase, Table 1) followed by Phase B, during which there were ships with hull-mounted MFAS as well as helicopter-dipping MFAS and active sonobuoys, which lasted three additional days. MdTag020’s tag stopped transmitting at 12:20 on 20 Aug, shortly after the end of Phase B, while MdTag021’s tag continued to transmit an additional 5 days until 18:52 on 24 Aug. Based on the modelled tag locations, the two animals appeared to remain together for the 6 days prior to the onset of hull-mounted MFAS.

In 2022, the typical activities of Phase A and B were mixed together in both training phases; therefore, the first week was called the mixed Phase A/B and the second week was called the mixed Phase B/A (Table 1). These lasted 3.5 days and 27 h, respectively, with a 3.5-day Interphase. The longest duration tag was deployed on 17 Aug 2022 at 14:09 on MdTag022 during the mixed Phase A/B and continued transmitting for 24.3 days until 10 Sept at 21:58. In contrast to the whale from 2014, the latter three Blainville’s beaked whales remained on or relatively near the range for the duration of their tag deployments (Fig. 1), even during the SCC with active MFAS.

In the movement model comparison, the CTCRW model was the best fit (i.e., had the lowest AIC score) for MdTag017 and MdTag022, while the BM model was the best fit for MdTag020 and MdTag021 (Table 2). All of MdTag017’s imputed tracks were rerouted around the 300 m isobath at Ka’ula Island, while a few of MdTag021’s imputed tracks were rerouted around the 300 m isobath west of Kaua’i (Fig. 1). Otherwise, all remaining tracks were deeper than 300 m and thus did not need to be rerouted.

**Table 2:** Comparison of movement models fitted to location data from tagged Blainville's beaked whales

Tag ID	Model AIC	
	CTCRW	BM
MdTag017	**3267.60**	3285.15
MdTag020	4950.95	**4948.49**
MdTag021	6783.62	**6781.61**
MdTag022	**16890.91**	16893.33

Highest ranked models based on AIC are bolded; imputed tracks derived from these best fit models were used for subsequent analyses. CTCRW = continuous time correlated random walk; BM = Brownian movement

### Dive behavior

Dive behavior data for the two 2021 deployments passed all quality control assessments and were used for analysis. MdTag020 conducted 175 dives, 45 (26%) of which were deep dives, and 130 (74%) of which were intermediate dives. MdTag021 conducted 249 dives, 59 (24%) of which were deep dives and 190 (76%) of which were intermediate. See supplemental material 1 for additional details on dive statistics. Since MdTag020 and MdTag021 remained in a group together until Phase B, their dive data were very similar. In total, 41 synchronous deep dives were performed by these two animals over 6 days. For two deep dives, the animals dove to the same depth, while in most dives they were separated by 16 to 352 m (mean 98.3 m) while at the deepest point.

IDDIs that included at least one intermediate dive ranged in duration from 18.3 to 291 min (mean 115.6 min) and contained up to 12 intermediate dives (Table 3). There were also two brief IDDIs between two consecutive deep dives that included unusual 300–500 m deep intermediate dives. MdTag020 also had a 131.5 min IDDI where they remained within 50 m of the surface during which MdTag021 conducted one intermediate dive. Finally, both whales had a 123-min IDDI where both animals remained within 50 m of the surface the entire time.

**Table 3:** Dive metrics for the combined dive record of MdTag020 and MdTag021

	Median Duration (min)	Minimum duration (min)	Maximum duration (min)	Median depth (m)	Minimum depth (m)	Maximum depth (m)	Minimum intermediate dive count	Maximum intermediate dive count
Deep dive	50.7	13.8	71.0	1200	304	1424	–	–
IDDI	112.5	0.5	291.0	104	–	–	1	12
Shallow dive	10.7	0.8	26.7	105	50	256	–	–

IDDI is the inter-deep dive interval. Deep dives were > 300 m, Intermediate dives were ≤ 300 m

### GVPs and tag-recorded dives

For the 9 and 13.3 days of data from MdTag020’s and MdTag021’s tags, respectively, only 29 dives met the dive spatial and temporal criteria and had start and end times that matched well with the GVPs. Of those, 23 dives from each tag started prior to the onset of the GVP detections (one of each were missing in the dive record for the other whale), and the GVP detections ended before the tagged animals surfaced. While this is not conclusive evidence that the GVPs match the dives of the tagged whales, it is highly likely to be the case given the spatial and temporal overlap (Supplemental Table S1).

Of those likely matching dives, echolocation pulses were detected at a median of 5.8 min (range 1.1–8.5 min, SD = 1.5) after the whales dove, starting at estimated depths of 70–646 m (median = 440 m, SD = 130 m; Table S1). The echolocation pulses ceased 23.4–3.1 min (median = 12.7, SD = 4.8 min) before the animals surfaced. The clicks ended at depths from the maximum depths of some dives up to 254 m (median 912 m, SD = 288 m). These dives were detected on 1–4 hydrophones per dive, and 8 times occurred subsequently on the same primary hydrophone or set of hydrophones indicating repeated diving at the same location.

### MFAS exposures and behavioral response analyses

#### Estimated received levels

All three of the Blainville’s beaked whales that overlapped spatially and temporally with SCCs had similar exposure paradigms and RLs. MdTag020 and MdTag021 were exposed to 15 bouts of MFAS, ranging in duration from 2 min to 2.21 h, while MdTag022 was exposed to 13 bouts of MFAS, ranging in duration from 1 min to 2.3 h. All three whales were on the south-western edge of the range when MFAS began, and all moved away from the centroid area of MFAS activity by 17.5–70.3 km.

**Fig. 2:**
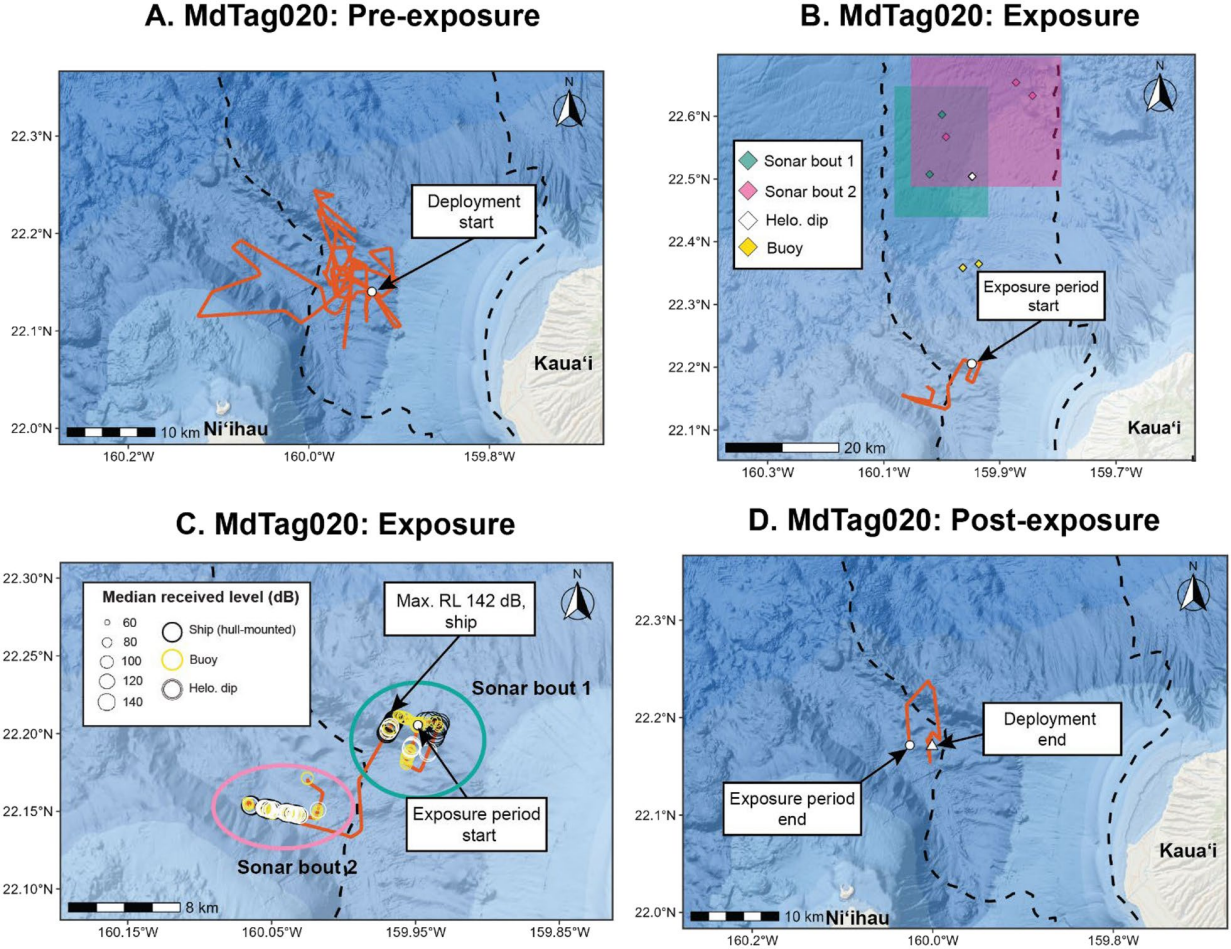
Movements of MdTag020 from the time of tag deployment until the start of MFAS given in panel A; the period during the two bouts of MFAS are given in panels B and C, with a wide view of the range in panel B, and a close up view of the track with corresponding median RLs shown during the bouts of MFAS in panel C; and the time from the end of the exposure periods to the end of the tag deployment given in panel D. Two bouts of MFAS are shown in B and C; mean ship locations for blocks of sonar within each bout are shown in panel B, as well as closest helicopter-dipping and active sonobuoy MFAS positions. PMRF is outlined by the dashed black line

**Fig. 3:**
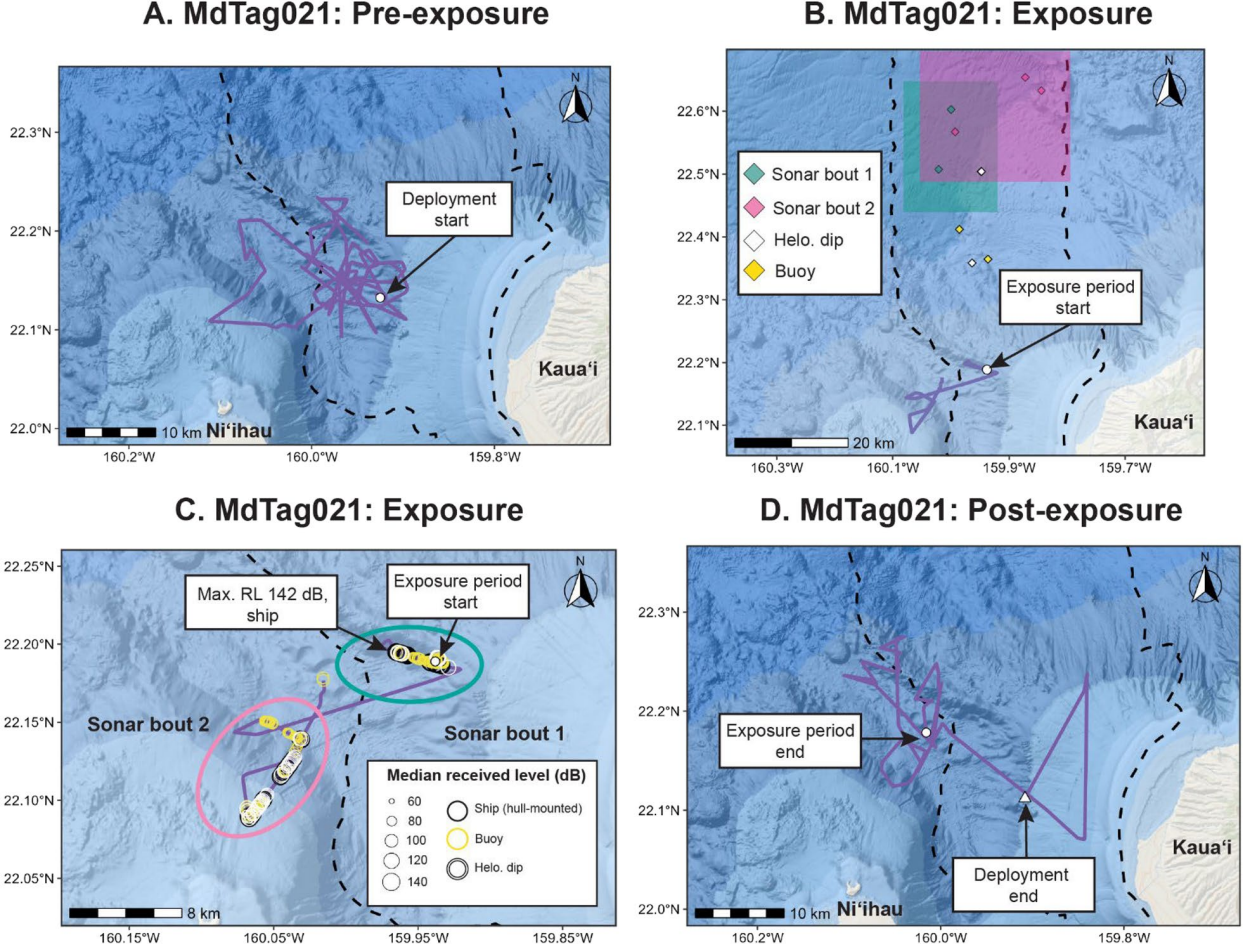
Movements of MdTag021 from the time of tag deployment until the start of MFAS given in panel A; the period during the two bouts of MFAS are given in panels B and C, with a wide view of the range in panel B, and a close up view of the track with corresponding median RLs shown during the bouts of MFAS in panel C; and the time from the end of the exposure periods to the end of the tag deployment given in panel D. Two bouts of MFAS are shown in B and C; mean ship locations for blocks of sonar within each bout are shown in panel B, as well as closest helicopter-dipping and sonobuoy MFAS positions. PMRF is outlined by the dashed black line

MdTag020 and MdTag021 were exposed to all three MFAS sources analyzed in this effort (hull-mounted, helicopter-dipping, and sonobuoy). The first exposures were from sonobuoys on 17 Aug 2021 at a median distance of 18 km, with estimated RLs of 73–105 dB re 1 µPa (Supplemental Figs. S1 and S2). Distances to sonobuoys increased for these first sonobuoy pings, indicating the animals were moving away from the MFAS, even though levels were lower than 105 dB re 1 µPa. However, the whales were on a foraging dive at the time that appeared to continue based on the continued detection of clicks with dive metrics that fell within fell within pre-MFAS parameters (see Dive Behavioral Response Analysis). Additional sonobuoy exposures occurred a few hours later at levels < 80 dB re 1 µPa, at the same time as the onset of hull-mounted MFAS. Most sonobuoy exposures occurred at distances of 35 to 55 km and RLs of 53 to 90 dB re 1 µPa (See Supplemental Figs. S1 and S2). The first helicopter-dipping sonar had a median RL of 114 dB re 1 µPa; additional helicopter-dipping MFAS exposures had median RLs of 101–122 dB re 1 µPa. The closest distance for helicopter-dipping sonar was 40 km. Hull-mounted MFAS exposures began on 17 Aug 2021 at around 135 dB re 1 µPa, with additional exposures on 18 and 19 Aug. Hull-mounted MFAS distances ranged from 19.5 to 60 km, with maximum median estimated RLs ranging from 124 to 146 dB re 1uPa. The whales were on the range for the first exposures to sonobuoys and helicopter-dipping MFAS and for the first hull-mounted MFAS exposure but then moved off the range for the second bout of MFAS (Fig. 3 and Fig. 2). MdTag020 remained just off the western edge of the range for the duration of their tag deployment; the tag remained attached to MdTag021 longer, so it can be observed that they moved back onto the range after the SCC (Fig. 3).

**Fig. 4:**
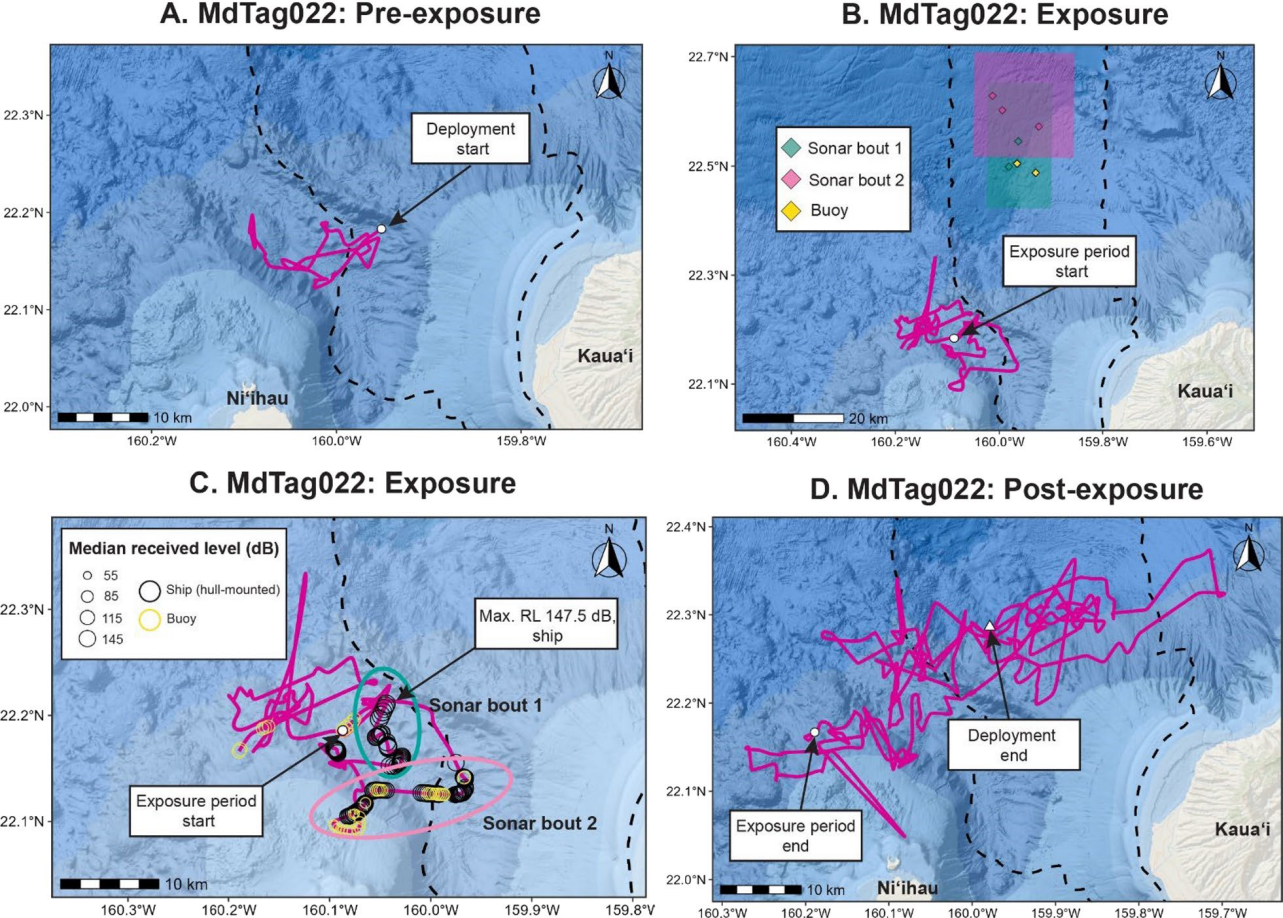
Movements of MdTag022 from the time of tag deployment until the start of MFAS given in panel A; the period during the two bouts of MFAS are given in panels B and C, with a wide view of the range in panel B, and a close up view of the track with corresponding median RLs shown during the bouts of MFAS in panel C; and the time from the end of the exposure periods to the end of the tag deployment given in panel D. m the time of tag deployment until the start of MFAS in panel A, during the two bouts of MFAS (wide view of range given in panel B, close up view of tracks with corresponding median RLs shown during the bouts of MFAS details given in panel C); and from the end of the exposure periods to the end of the tag deployment in panel D. Two bouts of MFAS are shown in B and C; mean ship locations for blocks of sonar within each bout are shown in panel B, as well as closest active sonobuoy MFAS positions. PMRF is outlined by the dashed black line

MdTag022 was exposed to both hull-mounted and sonobuoy MFAS in Aug 2022. The initial brief exposure was to an active sonobuoy on 19 Aug during Phase A/B, while the initial exposure to the first bout of hull-mounted MFAS began a few hours later and lasted for 20 min (Fig. 4). This period had the highest estimated median RLs between 144 to 148 dB re 1 µPa with relatively low standard deviations (e.g. good positional data with small error ellipses; Supplemental Fig. S3). Distances for these first exposures were estimated to be about 34 km. Exposures to sonobuoys occurred at distances between 27 and 67 km, including 95% CI positions; RLs were much lower than those of hull-mounted MFAS, with no sonobuoy MFAS median RL exceeding 100 dB re 1 µPa. MdTag022 had already moved just off the range to the west, to a very similar area to what MdTag020 and MdTag021 occupied in 2021, when the first bout of MFAS began (Fig. 4). They remained generally in this area for the rest of Phase A/B, the Interphase, and Phase B/A, although they did move further west at one point and then further south, so the MFAS bouts during Phase B/A had lower RLs. This animal also returned to the range after the SCC.

### Dive behavioral response analysis

**Fig. 5:**
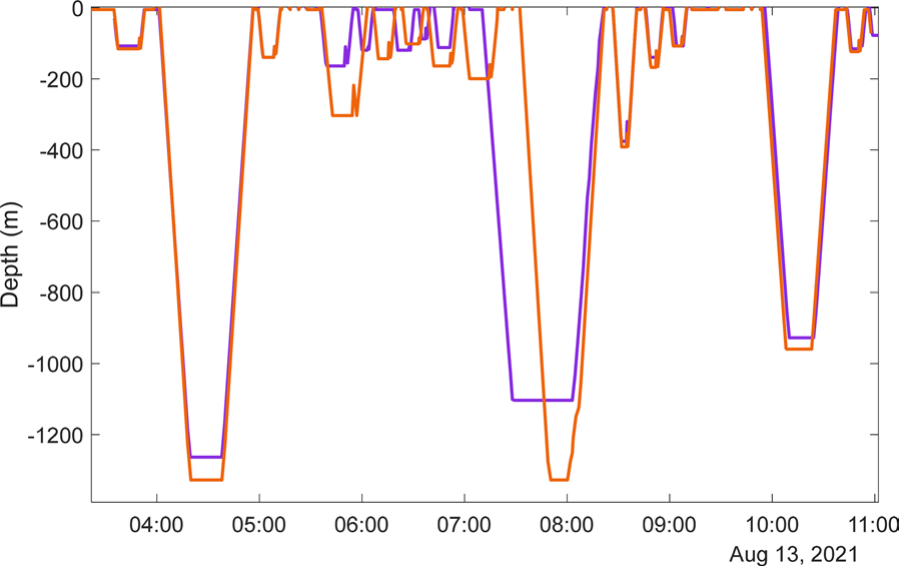
Modeled dive profiles of MdTag020 (orange) and MdTag021 (purple) during Phase A for the 2-h period the dives became asynchronous. MdTag020 conducted an anomalously deep intermediate dive to 303.5 m at the start of the asynchrony, and both whales conducted an anomalously deep intermediate dive to 375.5–391 m. Time on the x-axis is in HST

The first potential response to training activity occurred during Phase A, when the only two non-synchronous dives occurred prior to the onset of MFAS. The first of these was on 11 Aug at 19:15 shortly after the start of Phase A. Both animals went on a deep dive within a minute of each other, but MdTag020 surfaced first only to immediately dive again to the same depth as MdTag021; both animals then surfaced together. The second instance included two anomalous intermediate dives to greater than 300 m. Both animals had completed a deep dive on 13 Aug, with a subsequent intermediate dive (Fig. 5). Both animals dove again within a minute of each other, but MdTag020 dove to 303.5 m while MdTag021 only dove to 163.5 m. After this the whales conducted a series of asynchronous intermediate dives (Fig. 5), culminating in a deep dive that started at different times. However, both animals surfaced synchronously after this dive, then dove together and performed the second anomalous intermediate dive together to 375.5–391.5 m after which they surfaced together again. Their dive synchrony was restored after this series of dives to once again diving and surfacing within a few minutes of each other for most of the remainder of the shared dive record until the onset of hull-mounted MFAS.

**Fig. 6:**
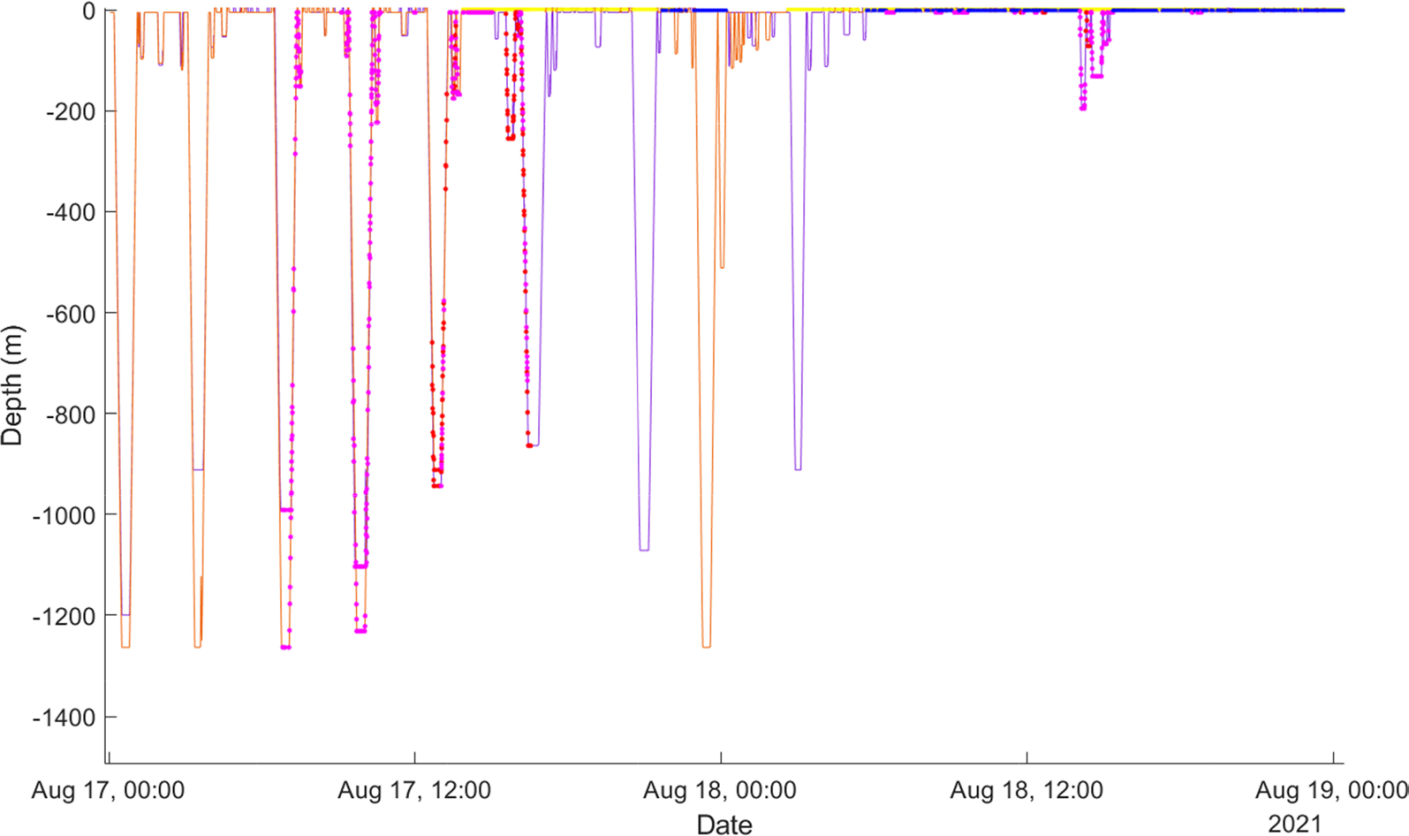
Dive record of MdTag020 (orange) and MdTag021 (purple) starting 17 August 2021 0:00 through 19 August 2021 12:00. The yellow bar at the surface indicates periods of missing data for MdTag020, the blue bar is missing data for MdTag021. Pink dots are 5 min bins with sonobuoy or helicopter dipping MFAS exposures, red dots are 5 min bins with hull-mounted MFAS exposures. Time on the x-axis is in HST

During Phase B, the first exposures for MdTag020 and MdTag021 were to sonobuoys dropped about 18 km away with median RLs generally below 100 dB re 1 µPa. As depicted in Fig. 6, these whales were together and at the bottom of a deep foraging dive when this first bout of MFAS began. This dive matched to a GVP on the range and was a full foraging dive with no cessation of clicking. No apparent response or change in dive behavior was observed based on the data available and the percentile evaluation of dive parameters.

The sonobuoy exposure continued through the subsequent intermediate dive, followed by a break in MFAS while the animals were at the surface. Then the whales conducted another deep foraging dive during a second bout of MFAS that included MFAS from both sonobuoys and helicopter dips, although RLs were still low (Supplemental Fig. S2, S3, S4). This dive may have matched a GVP on the range but started at the same time as the foraging clicks which would be unusual (clicks started at 19:24:51, tag dives start at 19:25:02 and 19:25:12), although other animals in the group could have dived sooner. Alternately, an early onset of clicks could also possibly indicate a vocal response to maintain coordination.

Finally, the third bout of MFAS began when the whales were about 707 m deep during their descent to what would be a 911.5 m dive, which was considerably shallower than the previous five foraging dives but within the range of normal dive depths (Fig. 6). This was the first exposure to hull-mounted MFAS, and as this dive also matched a GVP it appears they continued to forage throughout the dive, which lasted 50.0 min. Following this deep dive, the whales conducted 2 intermediate dives, stayed within the top 50 m for 79 min, then conducted 3 more intermediate dives for a total IDDI lasting 170.7 min. During this IDDI the pair moved off the range, however their interpolated tracks began to differ at this time so they may have split up. MdTag021 then conducted a deep dive to 863.5 m for 59.1 min (Fig. 6), but it is unknown whether MdTag020 conducted this dive as their dive record is missing at this time. Furthermore, because this deep dive was off the range it is unknown whether it was an actual foraging dive or not. Based on their subsequent dive and movement behavior (see the next section) the whales are likely separated at this point, as both whales do conduct another deep dive each but at different times and to different depths, and their predicted tracks appear to no longer be aligned, although since the tracks are smoothed this cannot be confirmed. During this time between bouts of MFAS, and immediately after the last deep dive recorded for MdTag020, she conducted her final anomalously deep intermediate dive to 511.5 m (Fig. 6). The dive record for both whales becomes intermittent at this point, so it can’t be said conclusively that they weren’t diving together; only that the final period of intermediate dives by both animals on 18 August is completely out of sync.

Most of the deep foraging dive metrics during and after the exposures fell between the 25th and 75th percentiles of the baseline dive data (Table 3). Two deep dives were slightly longer in duration, but shorter than the 97.5th percentile duration. The deep dive at the onset of hull-mounted MFAS was shallower than the 25th percentile depth but deeper than the 2.5th percentile depth. There was one long IDDI (202 min) after the longest (59.1 min), shallowest (863.5 m) deep dive with MFAS by MdTag021 (MdTag020 did not have dive data at the time), but all three metrics were still within the 95th percentile of baseline behavior.

In contrast, metrics were higher than the 75th percentile for many of the IDDI metrics during and after bouts of MFAS (Table 4). Note that the three anomalous intermediate dives > 300 m were not included in this analysis. Mean intermediate dive durations were longer than mean values from pre-MFAS data, with longer mean intermediate dives than the 75th percentile in four out of five periods during or after bouts of MFAS for MdTag021. Mean intermediate dive depths were over the 75th percentile range of pre-MFAS data during two of the three IDDIs for MdTag020 and in two of the five IDDIs for MdTag021. Similarly, the maximum intermediate dive depths were generally deeper during and after bouts of MFAS. In the same two IDDIs for MdTag020, the maximum intermediate dive depths exceeded the 75th percentile. This effect was even stronger for MdTag021, where in four of the five IDDIs the maximum intermediate dive depths exceeded the 75th percentile of baseline dives and one exceeded the 97.5th percentile. The results of the diel dive behavior analysis are included in Supplemental Materials (Tables S2–S3, Fig. S4).

**Table 4:** (A) percentile values of dive metrics for MdTag020 and MdTag021 during baseline and before MFAS bouts. (B) Actual metrics for the five dive cycles during bouts of MFAS

A		Deep dive duration	Deep dive depth	(IDDI) duration	# Int. dives following	Mean int. dive duration	Mean int. dive max depth	IDDI max depth
2.5%	MdTag020	17.3	349.7	3.2	0.0	4.0	56.7	62.0
	MdTag021	35.9	830.5	39.0	0.0	4.9	51.3	51.3
25.0%	MdTag020	47.4	955.5	74.7	2.0	8.3	97.5	119.5
	MdTag021	46.8	991.5	81.4	1.5	8.7	76.5	107.1
50.0%	MdTag020	51.1	1263.5	108.6	3.0	10.3	110.3	135.5
	MdTag021	50.3	1103.5	110.6	3.0	10.3	101.5	123.5
75.0%	MdTag020	54.6	1327.5	137.8	4.0	11.2	130.8	163.5
	MdTag021	53.0	1231.5	132.1	4.0	11.4	117.5	147.5
97.5%	MdTag020	59.2	1389.9	256.9	7.7	15.9	193.8	225.8
	MdTag021	61.3	1327.5	237.1	8.4	14.1	140.3	209.0

B		Exposure deep dive duration	Exposure deep dive depth	Exposure IDDI duration	Exposure # int. dives	Exposure mean int. dive duration	Exposure mean int. dive max depth	Exposure IDDI max depth
8/17/21 16:29	MdTag020	53.43	**1263.5**	122.20	3	8.19	96.83	151.5
	MdTag021	51.87	991.50	122.17	3	8.39	88.83	123.5
8/17/21 19:25	MdTag020	**55.57**	1231.5	128.37	2	10.80	**136.50**	**223.5**
	MdTag021	**55.67**	1103.50	128.33	2	**13.70**	117.50	**183.5**
8/17/21 22:29	MdTag020	50	**911.5**	30	2	11.05	**171.5**	**175.5**
	MdTag021	50.03	**943.50**	170.70	5	**11.71**	**139.10**	***255.50***
8/18/21 2:09	MdTag020	–	–	–	–	–	–	–
	MdTag021	**59.07**	**863.50**	**201.97**	3	**12.43**	**121.50**	**171.50**
8/19/21 0:04	MdTag020	–	–	–	–	–	–	–
	MdTag021	NA	NA	75.63	5	**12.98**	105.10	**195.5**

Note that MdTag020 is missing data for the last two bouts of MFAS, and that the final bout for MdTag021 is missing data before and after that period, so the IDDI data may be truncated. Values outside of the 25th to 75th percentiles of baseline data are highlighted in bold, and the values outside of the 2.5 or 97.5th percentiles are also italicized

### Movement behavioral response analysis

Movement data were available for MdTag017 for their full Baseline tag duration, 8.2 days. MdTag020 and MdTag021 generally remained associated over their overlapping period of tag attachment until the onset of hull-mounted MFAS, as discussed above. Information was available on movement patterns for Before (0.3 and 0.2 days, respectively), Phase A (1.7 days), the Interphase (3.8 days), Phase B (2.4 days) and the After period (0.9 and 5.2 days, truncated to 3 days for analysis). Both individuals remained on or in proximity to the range throughout the duration of the SCC. Information on movement patterns for MdTag022 in 2022 was available for the A/B Mixed Phase (3.4 days), the Interphase (3.5 days), the B/A Mixed Phase (1.2 days), and the After Phase (17.3 days, truncated to 3 days). The After data were truncated to 3 days to keep the time period approximately equivalent to the time periods of the other phases for statistical analyses.

**Fig. 7:**
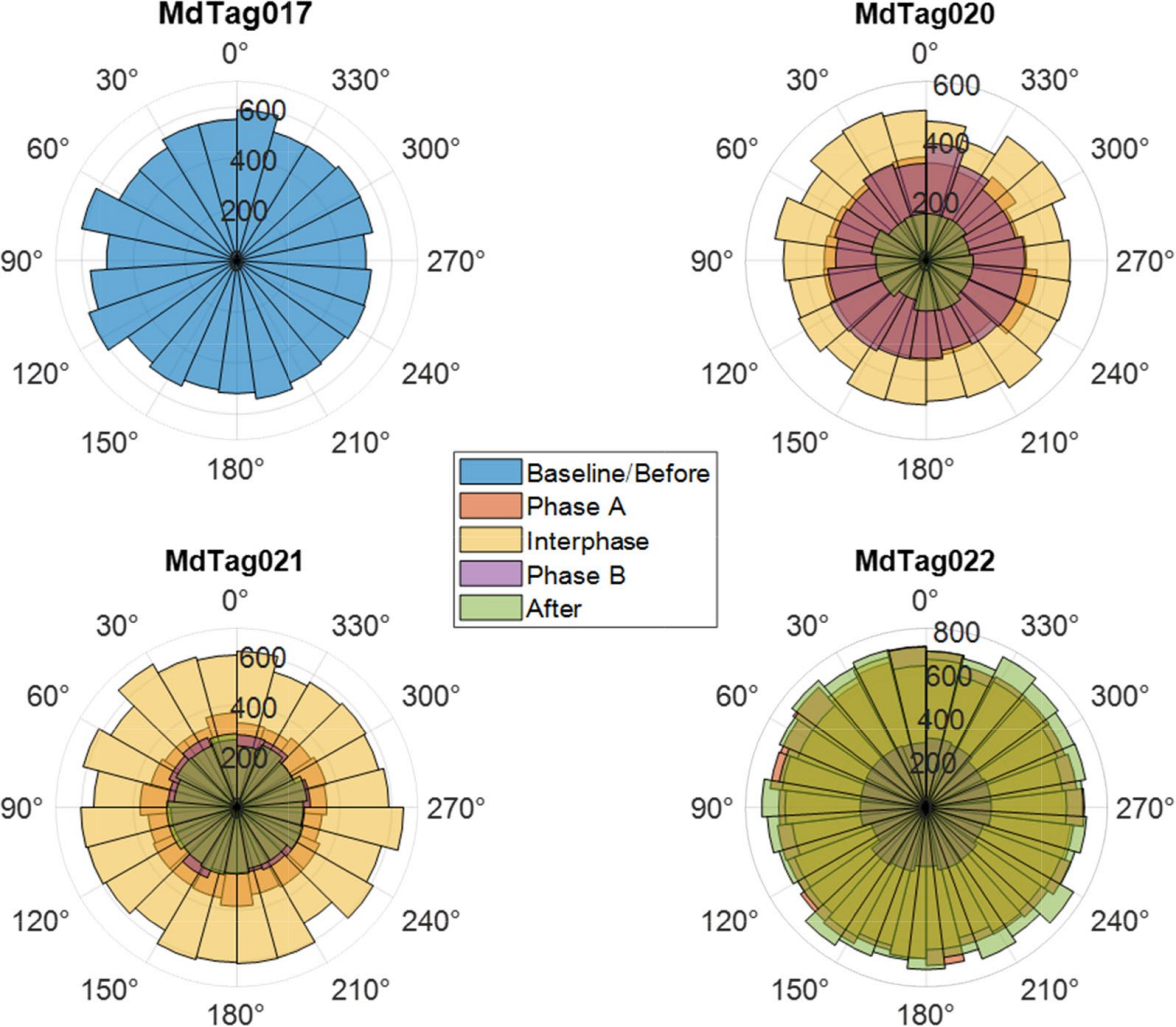
Track bearing values between 5 min predicted track steps, with all of MdTag017 being baseline data, while the data for the other three whales is broken down by Before, Phase A, Interphase, Phase B, and After periods. The numbers from 200 to 800 represent the number of 5 min predicted locations per phase

**Fig. 8:**
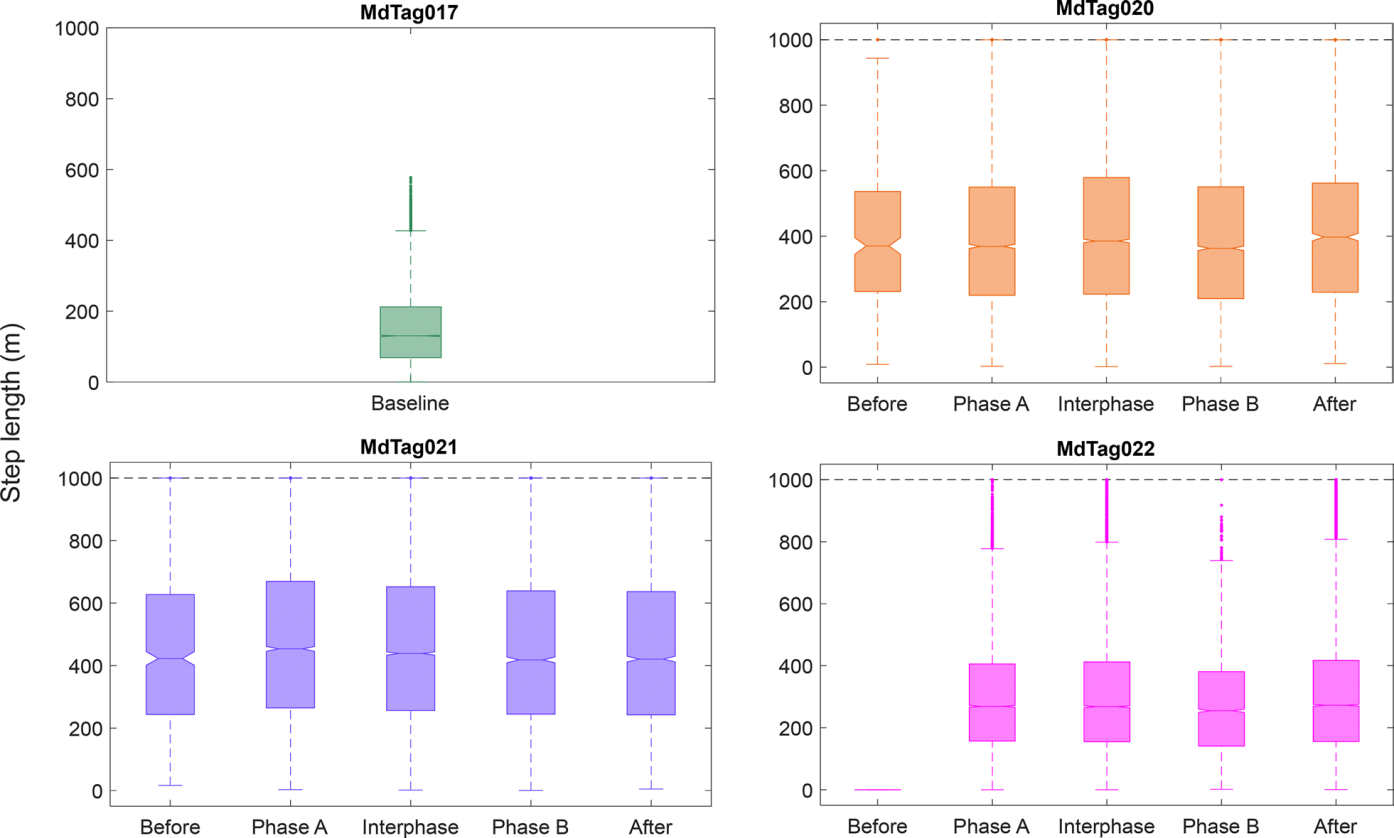
Track step length values between 5 min predicted track steps, with all of MdTag017 being baseline data, while the data for the other three whales is broken down by Before, Phase A, Interphase, Phase B, and After periods. Note that the y-axis has been constrained to 1000 m

**Fig. 9:**
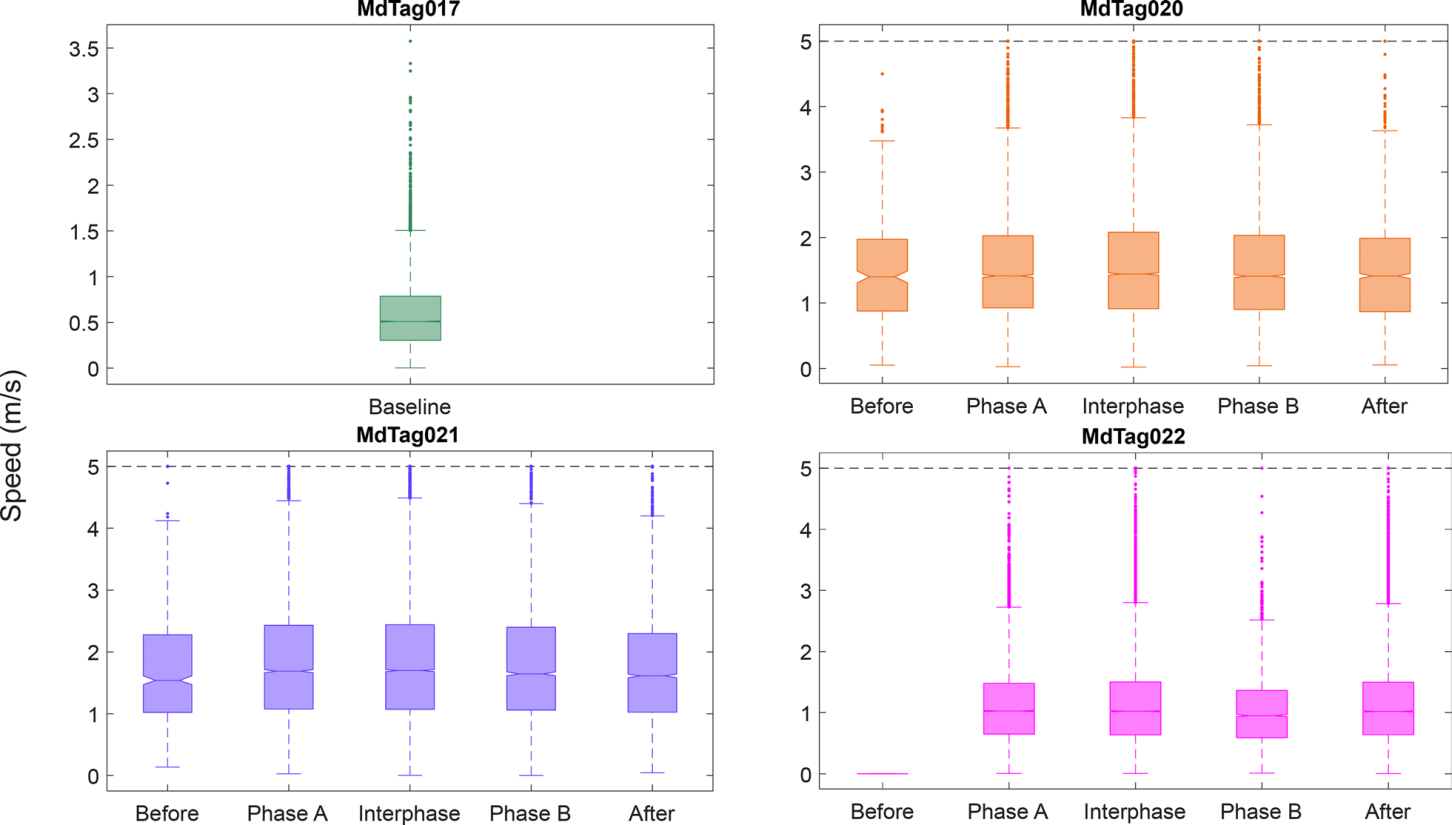
Track speed values between 5 min predicted track steps, with all of MdTag017 being baseline data, while the data for the other three whales is broken down by Before, Phase A, Interphase, Phase B, and After periods. Note that the y-axis has been constrained to 5 m/s

**Fig. 10:**
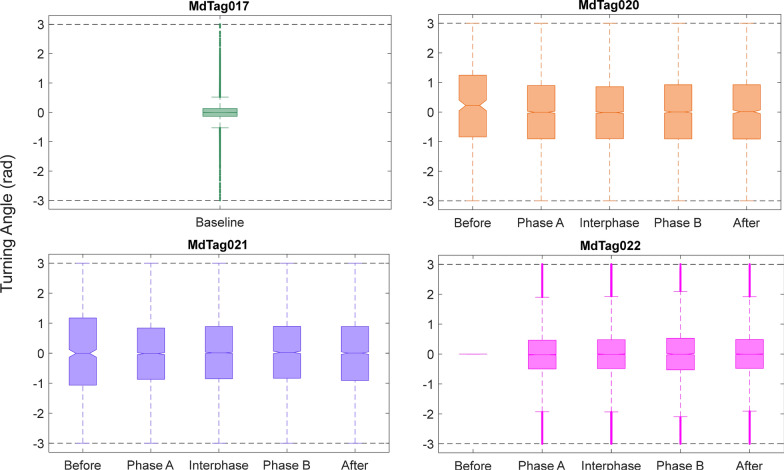
Track turning angle values between 5 min predicted track steps, with all of MdTag017 being baseline data, while the data for the other three whales is broken down by Before, Phase A, Interphase, Phase B, and After periods. Note that the y-axis has been constrained from −3 to 3

The movement variables of bearing, track step length, track speed, and turning angle were compared between the full 30 imputed tracks and tracks that only included predicted data within timestamps of original GPS or Argos locations less than 1 h apart. While the magnitude of the Chi-square and *p* values changed from the full tracks to the tracks with less than an hour between positions, the values that were significant remained so for all paradigms, as did the patterns in behavioral changes. Therefore, only the results of the 30 tracks with 1 h or less between inter-positional updates were included herein (Table 5, Figs. 7, 8, 9, 10). The Kruskal–Wallis results for the full tracks are included in supplemental materials (Table S4).

**Table 5:** Chi-square (top) and *p* values (bottom) from the Kruskal–Wallis tests of track movement variables

	MdTag020	MdTag021	MdTag022
Bearing (deg)	33.8	93.5	58.3
	** < 0.001**	** < 0.001**	** < 0.001**
Step length (m)	29.5	38.5	73.3
	** < 0.001**	** < 0.001**	** < 0.001**
Speed (m/s)	9.9	45.5	87.1
	**0.04**	** < 0.001**	** < 0.001**
Turning angle (rad)	5.1	2.7	6.4
	0.28	0.61	0.09

Track movement variables were compared across the SCC Phases Before, Phase A, Interphase, Phase B, and After for tracks with location intervals less than 1 h apart. Significant *p* values are in bold

The Before periods for MdTag020 and MdTag021 had little data once intervals between positions longer than 1 h were removed, and MdTag022 had no Before data; thus, MdTag017 becomes the best baseline for all the movement metrics. MdTag017’s mean step length was 130 m (SD = 105.04 m) with a mean speed of 0.5 m/s (SD = 0.37 m/s), and moved in a fairly directed manner with turning angles largely between −0.8 and 0.8 (SD = 0.42 rad) and movement in all directions. For the other whales, step length, speed, and bearing were all significantly different between at least two of the SCC phases (Table 5). To easily identify differences among each pair of phases, significance values for all Tukey–Kramer post hoc multiple comparison tests are given in Table 6.

**Table 6:** *P* values for the Tukey–Kramer post hoc multiple comparison test between SCC phases for each individual whale

		MdTag020				MdTag021				MdTag022			
		Bear	Step	Speed	Angle	Bear	Step	Speed	Angle	Bear	Step	Speed	Angle
Before	Phase A	**0.04**	0.99	0.98	0.34	0.99	**0.03**	**0.00**	0.95	–	–	–	–
Before	Interphase	**0.05**	0.91	1.00	0.39	0.99	0.31	**0.00**	1.00	–	–	–	–
Before	Phase B	0.57	0.98	0.85	0.23	**0.03**	0.99	**0.05**	1.00	–	–	–	–
Before	After	0.45	0.98	1.00	0.45	0.20	1.00	0.72	0.98	–	–	–	–
Phase A	Interphase	1.00	0.68	0.90	1.00	0.14	**0.05**	0.93	0.70	0.99	0.42	0.68	0.39
Phase A	Phase B	**0.00**	**0.01**	0.64	0.95	**0.00**	**0.00**	0.64	0.66	**0.00**	**0.00**	**0.00**	0.65
Phase A	After	0.18	0.18	0.57	1.00	**0.00**	**0.00**	**0.00**	1.00	**0.00**	0.12	0.99	0.06
Interphase	Phase B	**0.00**	**0.00**	0.13	0.85	**0.00**	**0.03**	0.17	0.99	**0.00**	**0.00**	**0.00**	1.00
Interphase	After	0.23	**0.02**	0.91	1.00	**0.00**	**0.01**	**0.00**	0.95	**0.00**	0.93	0.81	0.84
Phase B	After	0.96	1.00	0.98	0.96	**0.00**	1.00	**0.05**	0.89	**0.00**	**0.00**	**0.00**	0.97

Significant *p* values indicate differences between phases for each movement variable and are in bold

Travel direction (bearing) varied by phase for all three whales. For MdTag020 Phase A and Interphase were different from Before and Phase B; during Phase A and the Interphase they shifted their travel direction slightly more to the east. For MdTag021, both Phase B and After were significantly different from all other phases except Before, with travel more to the west-southwest during Phase B, and more to the east-northeast After. For MdTag022, Phase A/B and the Interphase were different than Phase B/A and After, with a shift in travel to the west for the latter two phases and most strongly in Phase B/A (Fig. 7, Table 6).

For MdTag020, step length was significantly shorter in Phase A and the Interphase than in Phase B; the step length during Interphase was also significantly shorter than After. In contrast, MdTag021 had significantly longer step lengths in Phase A than all other phases other than the Interphase, which also had significantly longer step lengths than in Phase B and After. Step length for MdTag022 was only significantly shorter during Phase B/A than the other phases (Fig. 8, Table 6).

Similar patterns occurred for the whales’ travel speed across phases. Once again, speed was highest for MdTag020 during Phase B and was lowest After, but not significantly so. MdTag021 had significantly higher rates of speed during Phase A, the Interphase, and Phase B than Before or After, although speed began decreasing during Phase B. Speed followed the same pattern for MdTag022 as their step length, with speed during Phase B/A significantly lower than all other phases (Fig. 9, Table 6).

Turning angle did not differ significantly across the phases for any of the whales, although travel did become slightly more directed for MdTag020 and MdTag021 after the Before phase, and slightly more directed for MdTag022 during Phase A/B (Fig. 10).

## Discussion

Our results provide insights into how Blainville’s beaked whales respond to MFAS at PMRF. While the sample size is small, tag data from these animals provide the first information on movement and dive behavior of Blainville’s beaked whales at PMRF during Navy training activity, and their relationship to passive acoustic data. In fact, this was the first time that movement and dives of satellite-tagged beaked whales were linked to acoustically detected GVPs, providing confirmation that the deep dives conducted by the beaked whales during at least the first three dives during MFAS exposures were still foraging dives. This sheds light on two long-held questions; first, do vocally active beaked whales that go quiet during MFAS leave the range or just stop foraging; and second, are satellite-tagged beaked whales (with no acoustic data) that conduct deep dives during exposures still foraging? These kinds of linked tag-acoustic studies on ranges can help fill in these data gaps, much like the use of sound recording and movement tags but over longer temporal scales.

Similar to the findings from beaked whales at other Navy ranges for both satellite-tagged animals (e.g., [49]) and acoustically detected animals (e.g., [55, 65, 76]), Blainville’s beaked whales on PMRF demonstrated some degree of temporary horizontal avoidance of training activities when exposed but remained within the area immediately surrounding the range. All three whales that were exposed to MFAS moved away from the training activity; at their furthest point the whales were 61.4 (MdTag020), 67.5 (MdTag021), and 48.9 km (MdTag022) away from the nearest active ship, and 20–48 km away from the nearest hydrophone on the range. However, all three animals remained in the Kaulakahi Channel between Ni’ihau and Kaua’i west of the range throughout the SCC. Furthermore, the habitat that the tagged whales utilized on and off the range was the same habitat where GVPs are typically detected [38], with steep slopes and water depths generally between 1500 and 2000 m. Previous observations of Blainville’s beaked whale GVPs before, during, and after SCCs have noted that GVPs are reduced both at the start of and throughout Phase A, with some recovery over the Interphase, and then are further reduced during Phase B [39, 44, 55]. This reduction in detected dives has now been directly linked with animals moving off the range rather than just ceasing to forage. Finally, these linked dive data also provide insight on the extended use of the range habitat by the same groups of whales. For example, this is the first time we can say with some certainty that repeated GVPs occurring on one or more hydrophones were in fact from the same group of animals. This kind of information can facilitate density estimation using spatially explicit capture-recapture methods, and we can begin to quantify repeated dives on-range by the same animals (e.g., [57]).

Received levels, distance to source, and source types have been used as explanatory variables to understand marine mammal behavioral responses to MFAS, and are often used as a means to compare results across different study paradigms and used in combined analyses such as the U.S. Navy’s behavioral risk functions [74]. In 2021, there were three sources of MFAS present during the SCC: hull-mounted, helicopter-dipping, and active sonobuoy. Although the sonobuoy exposures were closer in distance, their lower source level led to very low RLs, below 105 dB re 1 µPa at distances of 18 km or greater. The RLs in the first bout of helicopter-dipping MFAS in 2021 were slightly higher, up to median values of 122 dB re 1 µPa and a closest point of approach (CPA) of 40 km. During MdTag020’s and MdTag021’s third foraging dive during an exposure, hull-mounted MFAS began with median estimated RLs of up to 146 dB re 1 µPa and a CPA of 20 km, making this both the loudest and closest exposure to hull-mounted sonar for these whales. MdTag022 only had exposures to hull-mounted MFAS and active sonobuoys in 2022. This beaked whale was already to the west of the range when the exposures began, with the distance to the activity about 34 km away, and their initial exposure to hull-mounted MFAS resulted in median RLs up to 148 dB re 1 μPa. RLs from sonobuoys remained low throughout, with median levels never exceeding 92 dB re 1 μPa and remaining at distances of 27 km or more. Although there were some minor possible changes in dive behavior by MdTag020 and MdTag021 to the lower-level sources and they may have moved slightly away from the initial sonobuoy exposure, quantifiable changes in behavior did not begin until the onset of hull-mounted MFAS. In the previously mentioned controlled exposure experiment (CEE) studies, beaked whales responded to hull-mounted or simulated hull-mounted MFAS at RLs between 95 and 138 dB re 1 μPa and distances between 0.8 and 17 km [22, 73, 76, 78]. Therefore, while RLs may be comparable or higher to previous exposure levels that elicited responses, the distances were generally greater in the current study and may be a contributing factor as to why stronger responses were not observed. Further studies, ideally utilizing realistic, operational sources, contrasting distance and RLs in both animals that have a history of exposure and relatively naïve animals may help tease apart these explanatory variables. This would afford a better understanding of whether and how experience contributes to how an animal responds and better help regulators understand the conditions that lead to behavioral responses in areas of differential sonar activity.

By looking at simplified metrics of dive and movement behavior before, during, and after the different phases of the SCC, we can begin to detect behavioral responses by Blainville’s beaked whales. It should be noted that all three whales were tagged just before or during Phase A, during which there was training activity being conducted on the range. There were no MFAS sources present during Phase A in 2021, while during the 2022 SCC, the typical activity of Phase A and Phase B were mixed, therefore MdTag022 had MFAS exposures within 2 days of tag attachment. That said, there did not appear to be strong responses evidenced in the dive behavior of MdTag020 and MdTag021; while many of the dive metrics measured during the exposures were outside of the 75th (or 25th) percentiles of baseline data, only the maximum intermediate dive depth during one IDDI exceeded the 95th percentile of baseline data. This potential response was only observed for hull-mounted MFAS; there didn’t appear to be a dive response to the sonobuoy or helicopter-dipping MFAS exposures. The only other noticeable change in dive behavior were the two deeper intermediate dives that took place during Phase A and one during Phase B. However, dives to depths between 300 and 600 m are within the normal dive repertoire of Blainville’s beaked whales [8], and two of the three took place during the day when they have been typically documented [6].

In terms of the movement behavior, while the Before periods for MdTag020, MdTag021, and MdTag022 were either short or non-existent, the horizontal movement behavior of MdTag017 can provide a baseline of behavior. During the SCC, there were statistically significant differences in bearing, step length, and speed for all three beaked whales during Phase A, the Interphase, and Phase B. Interestingly, these metrics varied by individual, indicating there wasn’t a consistent response across all animals. For example, step length and speed were much lower for MdTag022 during Phase B than any other phase. These variables were also lower for MdTag020 during Phase A and the Interphase and then increased in Phase B and After, whereas for MdTag021 they had their highest speeds during Phase A and their lowest during Phase B and After. This seems counter-intuitive since these animals were together throughout the SCC until the start of Phase B, although it could be that step length and speed were comparable for MdTag020 and MdTag021 during Phase A and then changed in different directions once the animals split up, with MdTag020 moving faster and MdTag021 moving slower than their Phase A speed. However, the long dives of beaked whales limits the frequency of location transmissions, thus limiting our ability to capture their true continuous movements throughout the deployment. Location data from satellite-tagged beaked whales also tend to be characterized by lower location accuracy compared to other species (e.g., higher proportion of low-quality Argos class locations, see [69]); this is suspected to be in part due to thermal inertia in the tag resulting from deep, long-duration dives [43]. Consequently, increased positional error in a track can result in higher variability and uncertainty in predicted tracks. Furthermore, due to the frequent multi-hour intervals between GPS or Argos positional updates, the predicted track could become artificially variable during longer intervals, leading to possibly spurious metric values between locations and corresponding erroneous assumptions about the movement parameters during different phases. We attempted to account for this by removing track intervals with GPS or Argos updates separated by longer than 1 h and by running multiple imputations of the tracks, but the horizontal movement metrics computed here should be interpreted with a level of caution in consideration of the limitations of using smoothed tracks.

The analyses conducted here were deliberately simplified quantitative means of describing the data and characterizing possible changes in dive and spatial movement behavior across periods. In addition, there are many uncertainties within each step of the analyses; although they were addressed as much as possible, they do still lead to an unquantified amount of uncertainty in the results. As more data are obtained and more sophisticated methods are developed, more nuanced changes in behavior may be revealed. Nevertheless, these analyses indicated that the three Blainville’s beaked whales tagged at PMRF and exposed to MFAS did demonstrate some behavioral responses to the training activity including: slight changes in dive behavior; some changes in the direction and speed of travel; movement off the range into adjacent foraging habitat; and perhaps the strongest response, the separation of at least two individuals in a group after several days of remaining together and highly synchronized in their behavior (although it is possible that these animals separated for reasons other than disturbance as they had already been together for several days, and adult male-adult female Blainville’s beaked whales are not thought to have long-lasting social bonds; [6]). In previous CEEs on beaked whales, the most common reactions were a directed horizontal movement away from the source, and, if the animal was on a deep dive, a long, slow ascent from the dive [22, 64, 73, 76, 78]. In northern bottlenose whales (*Hyperoodon ampullatus*), the directed movement away from the source lasted for several hours, and the whales traveled continuously up to 37 km away from the exposure area [53, 78]. While the movement behavior herein does indicate changes in how the animals were moving, and all the animals did move away from the training activity, none of these whales demonstrated long lasting directed movement. As mentioned previously, in the CEEs in which a response was observed, the source was within 17 km and often within 3 km. When the source was further away, no behavioral responses were observed in goose-beaked whales (70–118 km; [22, 71]) or northern bottlenose whales (37–346 km; [78]). Therefore, it may be that the sources in this study were far enough away not to elicit a stronger movement response. Furthermore, median estimated RLs in this study never exceeded 150 dB re 1 μPa, which likely also contributed to a less intense response. Additionally, unlike the results found in the CEEs, there were no long, slow deep dives detected for MdTag020 or MdTag021; however, as these dives are modeled and not measured as with a DTAG, the longer duration dives could have had shallow and slow ascents that would not be captured. The CEE studies were conducted using DTAGs (e.g., [48]) that collect fine-scale movement and dive data and were able to record nuanced changes in dive behavior that satellite tags cannot. In addition, the dive records in this study were very sparse after the first three dives with MFAS, and so there may have been subsequent stronger changes in dive behavior that were not recorded. No previous CEEs have mentioned a group splitting apart as a response to MFAS, but this may be an artifact of most CEEs relying on fine-scale data from single animals. Recent studies of exposures to groups of goose-beaked whales have also found groups to split in response to MFAS [72].

While the movement behavior of MdTag017 can only act as baseline behavior, it is interesting to note that MdTag017 was the only animal tagged in February and that it moved well away from the range prior to the onset of MFAS. It is possible that this individual/group was part of an open-ocean population (see [9]), rather than an island-associated group, since the animals in that group had not been photographed before or since. These differences in habitat use could also be indicative of abundance and location of preferred prey at different times of year. This may begin to elucidate why animals remain in the area and presumably forage even during repeated periods of MFAS, if prey is concentrated in certain areas at different times of the year, in addition to the possibility of habituation to or tolerance of the MFAS signals. Thus, the lack of strong responses in this study may stem from a combination of environmental (prey availability, seasonality), internal (body condition, habituation), and external (distance to sources, low to moderate RLs) factors that differed from previous CEEs on beaked whales.

There are some similarities in the observed changes in dive behavior in this study to those found in another range-based opportunistic study of goose-beaked whales on SOAR that also looked at both hull-mounted and helicopter-dipping MFAS. In that study, data from 16 animals were aggregated to model changes in dive behavior relative to the different sources [28]. For the lower source level helicopter-dipping sonar, closer proximity of the source led to increased durations of both intermediate and deep dives, even more so than for hull-mounted MFAS, and dives were longest when both sources were present [28]. Both intermediate and deep dives were similarly slightly longer at PMRF during exposures to all source types (Table 4), but never exceeded the 95th percentile baseline values. In contrast to what was found at PMRF for Blainville’s beaked whales where intermediate dives during exposures were the deepest recorded, the goose-beaked whales on the SOAR range conducted shallower intermediate dives during MFAS exposures. Finally, both surface intervals and IDDIs were longer during exposures at SOAR, especially at closer distances [28]; in this study, IDDIs were only slightly longer for two of the dive cycles and only for hull-mounted MFAS. Interestingly, Falcone et al. [28] also observed four unusually brief anomalous intermediate dives, similar to the three intermediate dives recorded in this study. The goose-beaked whales were often much closer to the helicopter-dipping sonar at SOAR than the Blainville’s beaked whales in this study were to either helicopter-dipping or sonobuoy MFAS; this may be why a response was observed for the former population to that lower powered source and less so in the present study. Falcone et al. [27, 28] hypothesized that the stronger reactions observed for the lower powered source was due to the “random” nature of exposure, where the source appeared suddenly and then disappeared, unlike a ship that could be heard approaching or moving away. Future opportunistic studies at PMRF should continue with both beaked whales and other cetacean species with these additional sources of MFAS, in order to determine if closer proximity may elicit a behavioral response.

In addition to the information on Blainville’s beaked whale behavioral responses to MFAS at PMRF, this study also provides some new data on their baseline behavior and habitat use as well. For example, off Kaua’i, the median deep dive depth for Blainville’s beaked whales was 1113 m, the maximum depth was 1424 m, and the median deep dive duration was 51 min (range 14–71 min). Off the island of Hawai’i, Blainville’s beaked whales were recorded diving to median depths of 1099 m during the day (896–1409 m) and 1052 m (872–1182 m) at night for dives deeper than 800 m, with a maximum depth of 1599 m. Deep dive durations lasted a mean of 54.4 min (51–60 min) during the day and 51.3 min (43–58 min) at night, with maximum durations of 68 min during the day and 83 min at night [8]. These values are comparable to what was found off Kaua’i, although it makes sense that these parameters are likely to be similar in such spatially proximate populations, with variations likely due to differences in habitat at the two islands. The Blainville’s beaked whales off the island of Hawai’i also appear to be a resident population that co-occur with a resident population of goose-beaked whales [6, 63]. It appears the two species have partitioned their environment and prey resources, such that the goose-beaked whales dive more deeply and are found in deeper water depths (generally but not always further offshore), while the Blainville’s beaked whales occur in slightly more shallow waters [6]. Niche partitioning among beaked whales can also be assessed at PMRF; although goose-beaked whales have never been visually detected at PMRF [5] there are acoustic detections in deep water, along with Longman’s (*Indopacetus pacificus*) and the Cross Seamount beaked whale [56, 58, 62]. The spatial relationship and habitat use of the range complex can be examined for these species to determine if similar spatial partitioning is occurring off Kaua’i as was found off the island of Hawai’i.

Finally, multi-day dive synchrony was found in the pair of tagged Blainville’s beaked whales in this study. In goose-beaked whales, high levels of dive synchrony have been observed in male pairs for periods of days to weeks, while levels of synchrony between an adult male and an animal of another sex or age class were much lower [14]. In this case, MdTag020 was a female and MdTag021 a male, indicating that long duration associations with high dive synchrony can occur across sex classes in Blainville’s beaked whales. MdTag020 tended to dive deeper than MdTag021 on their synchronous dives, and often initiated a deep dive before MdTag021. Dive synchrony was also observed in another tagged male–female pair of Blainville’s beaked whales off the island of Hawai’i [6], with the female at different depths than the male on deep foraging dives as well as during the intermediate dives. Tight synchrony while diving but spatial separation at depth has been observed in other tagged pairs of both Blainville’s and goose-beaked whales [3, 4] and may prevent individuals in a group from competing for specific prey items while at depth.

Little is known about the population of Blainville’s beaked whales that occupy the waters off Kaua’i, but based on preliminary photographic evidence there may be a resident beaked whale population like that found off the island of Hawai’i. There are certainly Blainville’s beaked whales acoustically present year-round at PMRF so it is likely that members of the Hawaiian population as a whole may have been exposed at least once, and possibly multiple times, to MFAS that has occurred on or off the range, and an island-associated population even more so. If these animals are resident, they are likely exposed to Navy training activity and the use of MFAS with some regularity and may have habituated to the sounds. Alternately, foraging needs may drive their behavior regardless of anthropogenic activity [32], and therefore they continue to forage off the range during exposures and tolerate the sound because that is where their prey is concentrated. Additional tagging and photo-identification studies are critical for this population to understand their habitat use and residency in this area, and to be able to assess the potential impact of repeated exposures to MFAS. Prey mapping of the water column on and off the range would also provide insight into potential motivations and drivers of beaked whale behavior. However, these tag data, coupled with the linked GVPs on the range, begin to provide some of this insight into both baseline habitat use and behavioral response of potentially resident animals to Navy training and multiple sources of MFAS.

## Conclusions

Three of four Blainville’s beaked whales satellite-tagged at PMRF before SCC training events remained on or near the range during training activity that included MFAS from three sources. Mild behavioral responses in the form of dive metrics outside normal bounds and significantly different values in speed, step length, and bearing across phases were found, but this analysis was conducted on modeled dive data and interpolated track data, and so can only be taken as preliminary results. Although they moved off the range away from the MFAS sources in a similar manner as has been observed at other ranges, the whales remained within 10 s of kms of the range throughout the training, and the two whales with data still transmitting after the training returned to the range. RLs were low to moderate (up to 150 dB re 1 μPa) and distances to any of the sources were no closer than 18 km. These three whales, all tagged in August, may be part of a resident community of Blainville’s beaked whales that have been repeatedly exposed to MFAS and may be habituated or have learned to tolerate the sound, while the fourth whale, tagged in February, left the area before the onset of training activity, and may be part of an open-ocean population based on the lack of re-sightings of the group. It is also possible that prey resources are distributed differently in winter and summer around Kaua’i. Therefore, whether and to what magnitude a response may occur to MFAS at PMRF is likely related to multiple interacting factors, including residency, exposure history, season, the occurrence of multiple sources, proximity and relative movement of source(s), RL, prey distribution, and potentially other contextual factors. More work needs to be done to tease apart these factors to better understand why Blainville’s beaked whales at PMRF demonstrated a milder response to MFAS than beaked whales in other exposure studies have done.

## Supplementary Information

Supplementary Materials 1.

Supplementary Materials 2. Hawaiian abstract.

## Data Availability

Tag data is available on Movebank, https://www.movebank.org/ Modeled received level and source positional data cannot be made publicly available as they are classified.

## Abbreviations

AIC: Akaike’s information criteria
ANOVA: Analysis of variance
AUTEC: Atlantic Undersea Testing and Evaluation Center
BM: Brownian movement
CI: Confidence interval
CPA: Closest point of approach
CTCRW: Continuous time correlated random walk
GPS: Global positioning system
GVP: Group vocal period
HST: Hawaiian Standard Time
IDDI: Inter-deep dive interval
MFAS: Mid-frequency active sonar
NOAA: National Oceanic and Atmospheric Administration
PAM: Passive acoustic monitoring
PMRF: Pacific Missile Range Facility
RHIB: Rigid-hulled inflatable boat
RL: Received level
SCC: Submarine Command Course
SOAR: Southern California Offshore Acoustic Range
