# Supplementary material for: Exposure and response of satellite-tagged Blainville’s beaked whales to mid-frequency active sonar off Kaua‘i, Hawai‘i
Source: Mov Ecol. 2025 Apr 21;13:29. doi: 10.1186/s40462-025-00550-9 (PMC12010625; doi:10.1186/s40462-025-00550-9)
Supplement: Supplementary file 1 — Supplementary Materials 1. [file 40462_2025_550_MOESM1_ESM.docx]

# SUPPLEMENTAL MATERIAL

## Results of GVP and Dive Comparison

Table S1 - Tag dive start and end times compared against the acoustically detected Group Vocal Periods (GVPs) on the range.

|  | Date | Tag Dive Start Time | Tag Dive End Time | Max Dive Depth | GVP Start | GVP  End | Descent time diff | Ascent time diff | Click Start Depth | Click End Depth |
| --- | --- | --- | --- | --- | --- | --- | --- | --- | --- | --- |
| MdTag020 | 8/12/2021 | 10:37:46 | 11:25:50 | 1327.5 | 10:42:31 | 11:08:56 | 4.75 | 16.90 | 403.6 | 1327.5* |
| MdTag021 | 8/12/2021 | 10:39:04 | 11:26:20 | 1263.5 | 10:42:31 | 11:08:56 | 3.45 | 17.40 | 283.8 | 1263.5* |
| MdTag020 | 8/12/2021 | 13:19:40 | 14:07:02 | 1359.5 | 13:25:14 | 14:03:51 | 5.57 | 3.18 | 491.6 | 281.1 |
| MdTag021 | 8/12/2021 | 13:19:14 | 14:06:58 | 1327.5 | 13:25:14 | 14:03:51 | 6.00 | 3.12 | 513.4 | 266.7 |
| MdTag020 | 8/12/2021 | 21:49:08 | 22:40:26 | 911.5 | 21:56:39 | 22:22:51 | 7.52 | 17.58 | 410.9 | 911.5* |
| MdTag021 | 8/12/2021 | 21:49:42 | 22:40:54 | 959.5 | 21:56:39 | 22:27:11 | 6.95 | 13.72 | 400.8 | 790.9 |
| MdTag020 | 8/12/2021 | 23:21:00 | 0:07:16 | 927.5 | 23:28:42 | 23:54:54 | 7.70 | 12.37 | 617.4 | 927.5* |
| MdTag021 | 8/12/2021 | 23:22:24 | 0:07:52 | 959.5 | 23:28:42 | 23:54:54 | 6.30 | 12.97 | 531.8 | 959.5* |
| MdTag020 | 8/13/2021 | 4:52:54 | 5:44:36 | 1423.5 | 4:56:18 | 5:35:16 | 3.40 | 9.33 | 288.0 | 790.7 |
| MdTag020 | 8/13/2021 | -- | -- | -- | -- | -- | -- | -- | -- | -- |
| MdTag020 | 8/13/2021 | 8:06:54 | 8:54:12 | 1263.5 | 8:10:13 | 8:35:30 | 3.32 | 18.70 | 272.6 | 1263.5* |
| MdTag021 | 8/13/2021 | 8:04:54 | 8:53:52 | 1263.5 | 8:10:13 | 8:35:30 | 5.32 | 18.37 | 422.1 | 1263.5* |
| MdTag020 | 8/13/2021 | 22:18:26 | 23:06:20 | 911.5 | 22:26:55 | 22:55:18 | 8.48 | 11.03 | 645.7 | 839.8 |
| MdTag021 | 8/13/2021 | 22:19:24 | 23:07:08 | 911.5 | 22:26:55 | 22:55:18 | 7.52 | 11.83 | 574.1 | 903.9 |
| MdTag020 | 8/14/2021 | 8:14:44 | 9:04:44 | 1231.5 | 8:20:13 | 8:58:17 | 5.48 | 6.45 | 540.2 | 635.5 |
| MdTag021 | 8/14/2021 | 8:14:44 | 9:04:30 | 1135.5 | 8:20:13 | 8:58:17 | 5.48 | 6.22 | 500.4 | 567.4 |
| MdTag020 | 8/14/2021 | 11:16:06 | 12:08:50 | 1231.5 | 11:21:56 | 11:54:33 | 5.83 | 14.28 | 419.2 | 1026.3 |
| MdTag021 | 8/14/2021 | 11:16:00 | 12:08:44 | 1199.5 | 11:21:56 | 11:54:33 | 5.93 | 14.18 | 539.9 | 1199.5* |
| MdTag020 | 8/14/2021 | 16:00:26 | 16:59:14 | 1327.5 | 16:06:37 | 16:42:05 | 6.18 | 17.15 | 429.5 | 1191.3 |
| MdTag021 | 8/14/2021 | 16:00:52 | 16:59:16 | 1199.5 | 16:06:37 | 16:42:05 | 5.75 | 17.18 | 363.4 | 1086.0 |
| MdTag020 | 8/14/2021 | 18:27:10 | 19:18:18 | 1263.5 | 18:33:54 | 19:13:08 | 6.73 | 5.17 | 511.9 | 392.8 |
| MdTag021 | 8/14/2021 | 18:27:06 | 19:18:02 | 1135.5 | 18:31:02 | 18:58:23 | 3.93 | 19.65 | 269.8 | 1135.5* |
| MdTag020 | 8/15/2021 | 4:27:46 | 5:27:16 | 1359.5 | 4:34:47 | 5:08:45 | 7.02 | 18.52 | 493.3 | 1301.8 |
| MdTag021 | 8/15/2021 | 4:30:28 | 5:27:26 | 1199.5 | 4:34:47 | 5:08:45 | 4.32 | 18.68 | 279.7 | 1199.5* |
| MdTag020 | 8/16/2021 | 11:18:08 | 12:08:48 | 1359.5 | 11:23:45 | 11:56:29 | 5.62 | 12.32 | 463.7 | 1016.9 |
| MdTag021 | 8/16/2021 | 11:18:08 | 12:08:52 | 1327.5 | 11:23:45 | 11:56:29 | 5.62 | 12.38 | 452.2 | 997.0 |
| MdTag020 | 8/16/2021 | 13:20:50 | 14:13:46 | 1359.5 | 13:27:09 | 14:02:15 | 6.32 | 11.52 | 499.2 | 910.1 |
| MdTag021 | 8/16/2021 | 13:22:20 | 14:13:48 | 1295.5 | 13:27:09 | 14:02:15 | 4.82 | 11.55 | 485.0 | 1162.9 |
| MdTag020 | 8/16/2021 | 17:01:04 | 17:51:10 | 1039.5 | 17:05:57 | 17:41:02 | 4.88 | 10.13 | 311.8 | 646.9 |
| MdTag021 | 8/16/2021 | 17:04:52 | 17:51:30 | 975.5 | 17:05:57 | 17:41:02 | 1.08 | 10.47 | 69.7 | 673.7 |
| MdTag020 | 8/16/2021 | 21:39:12 | 22:26:08 | 1199.5 | 21:44:23 | 22:13:21 | 5.18 | 12.78 | 407.6 | 1005.3 |
| MdTag021 | 8/16/2021 | 21:40:18 | 22:26:32 | 1071.5 | 21:44:23 | 22:13:21 | 4.08 | 13.18 | 378.5 | 1071.5* |
| MdTag020 | 8/17/2021 | -- | -- | -- | -- | -- | -- | -- | -- | -- |
| MdTag021 | 8/17/2021 | 0:35:14 | 1:29:54 | 1103.5 | 0:39:29 | 1:06:30 | 4.25 | 23.40 | 264.0 | 1103.5* |
| MdTag020 | 8/17/2021 | 7:08:00 | 8:06:54 | 1295.5 | 7:12:55 | 8:03:09 | 4.92 | 3.75 | 332.7 | 253.8 |
| MdTag021 | 8/17/2021 | 7:13:38 | 8:06:28 | 1103.5 | 7:12:55 | 8:03:09 | 0.72 | 3.32 | 5 | 277.1 |
| MdTag020 | 8/17/2021 | 10:12:24 | 11:07:04 | 1263.5 | 10:19:05 | 10:54:25 | 6.68 | 12.65 | 475.3 | 899.6 |
| MdTag021 | 8/17/2021 | 10:12:34 | 11:07:14 | 1199.5 | 10:19:05 | 10:54:25 | 6.52 | 12.82 | 440.0 | 865.3 |
| MdTag020 | 8/17/2021 | 13:05:26 | 13:54:44 | 1263.5 | 13:11:29 | 13:42:41 | 6.05 | 12.05 | 477.1 | 950.2 |
| MdTag021 | 8/17/2021 | 13:05:16 | 13:54:44 | 911.5 | 13:11:29 | 13:42:41 | 6.22 | 12.05 | 458.2 | 888.2 |
| MdTag020 | 8/17/2021 | 16:29:12 | 17:22:38 | 1263.5 | 16:37:15 | 17:11:14 | 8.05 | 11.40 | 585.7 | 829.4 |
| MdTag021 | 8/17/2021 | 16:31:00 | 17:22:52 | 991.5 | 16:37:15 | 17:11:14 | 6.25 | 11.63 | 477.9 | 889.5 |
| MdTag020 | 8/17/2021 | 19:25:12 | 20:20:46 | 1231.5 | 19:24:51 | 20:07:13 | -0.35 | 13.33 | NA | 879.8 |
| MdTag021 | 8/17/2021 | 19:25:02 | 20:20:42 | 1103.5 | 19:24:51 | 20:07:13 | -0.18 | 13.29 | NA | 1036.6 |
| MdTag020 | 8/17/2021 | 22:29:00 | 23:19:00 | 911.5 | 22:35:54 | 23:02:00 | 6.90 | 17.00 | 387.0 | 911.5* |
| MdTag021 | 8/17/2021 | 22:28:20 | 23:18:22 | 943.5 | 22:35:54 | 23:02:00 | 7.57 | 16.37 | 439.0 | 943.5* |

## *Table includes estimates of the times when clicks started and stopped and the relative modeled depths at those times. Click end depths with an asterisk indicate that according to the modeled dive depths, at the time the clicks stopped the animals were still at their maximum dive depths. There is no dive data for one dive from MdTag020 and for one dive from MdTag021.*

## Modeled MFAS Received Levels


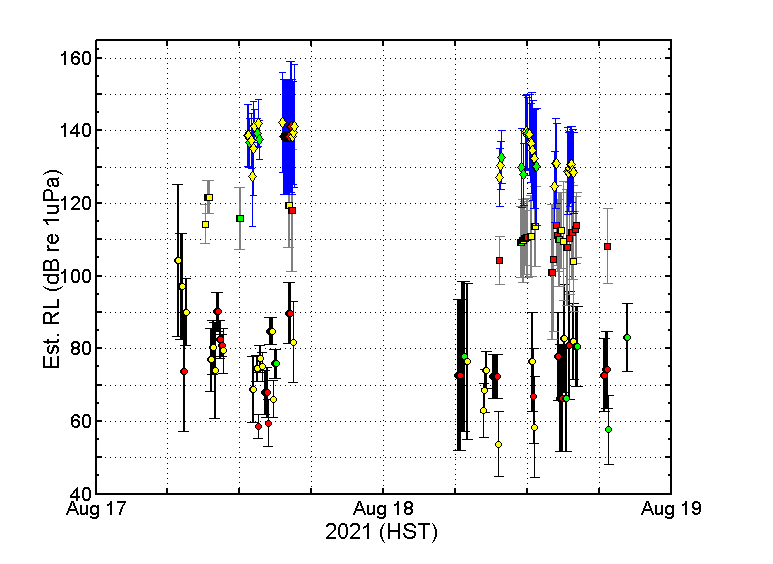


Figure S1 - Estimated RLs for hull-mounted MFAS (blue) and helicopter-dipping (grey) and sonobuoy MFAS (black) for MdTag020 provided as maximum median +/- 2 standard deviations in 5 min bins. The relative amount of sonar activity in each bin is indicated as a stoplight color coded symbol with green for lowest activity, yellow for moderate, and red for higher activity.


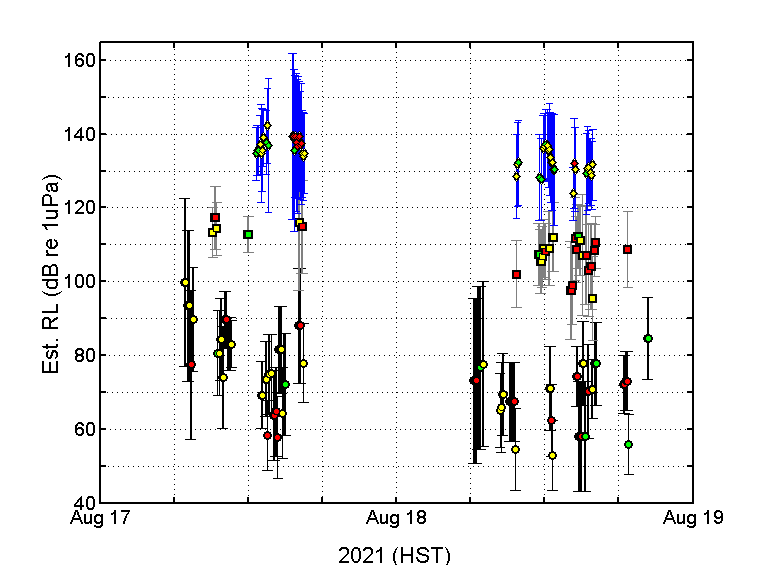


Figure S2 - Estimated receive levels for hull-mounted MFAS (blue) and helicopter-dipping and sonobuoy MFAS (black) for MdTag021 provided as maximum median +/- 2 standard deviations in 5 min bins. The relative amount of sonar activity in each bin is indicated as a stoplight color coded symbol with green for lowest activity, yellow for moderate, and red for higher activity.


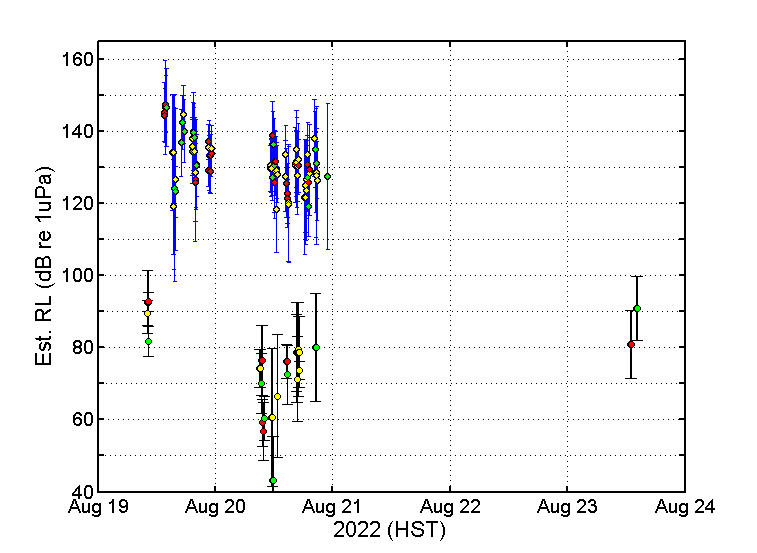


Figure S3 - Estimated RLs for hull-mounted MFAS (blue) and sonobuoy MFAS (black) for MdTag022 provided as maximum median +/- 2 standard deviations in 5 min bins. The relative amount of sonar activity in each bin is indicated as a stoplight color coded symbol with green for lowest activity, yellow for moderate, and red for higher activity.

## Diel Dive Analysis Methods and Results

To account for the impacts of potential diel patterns on diving behavior (see Baird et al. 2008), the coverage of each tag based on time of day for each SCC Phase was also calculated (Table SW2). Dive and surface periods were each assigned either as a day or night period based on when they started. The durations of surface periods that spanned more than one time of day were split. Surface periods that crossed multiple SCC phases had their durations split between phases. Due to their relatively short duration, no dives were split based on either phase or time of day. Coverage by time of day for each tag was calculated as the total duration of dive and surfacing periods within the time of day and SCC phase of interest, divided by the total duration of the time of day within that particular phase (e.g., the day total duration for Phase A would represent the sum of the duration of all days within Phase A). For the before and after periods, for all analyses only 3 days prior to the start of Phase A, and 3 days following the end of Phase B were used. Metrics that were calculated for phases and times of day with sufficient coverage included the dive rate (number of dives per hour), percentage of time spent at the surface, median dive depth, and median dive duration. Kruskal-Wallis one-way ANOVAs were used to identify significant differences in dive depth and duration among phases, and by night/day period, for the two whales with sufficient dive/surfacing coverage, and post-hoc Dunn’s tests with a Benjamini-Hochberg correction were conducted to identify phases where pairwise significant differences were detected (e.g., statistical difference between Phase A and B; significance level for both tests = 0.05). Sufficient coverage for each phase and time of day was defined as having dive behavior data available for at least 50% of the total duration of each phase within that time of day.

Table S2 - Behavior data coverage by SCC phase.

| **Individual** | **Percentage of dive/surfacing data** | | | | |
| --- | --- | --- | --- | --- | --- |
|  | **Before** | **Phase A** | **Interphase** | **Phase B** | **After** |
| **MdTag020** |  |  |  |  |  |
| Duration overall (days) | 0.3 | 1.7 | 3.8 | 2.4 | 0.9 |
| Days dive/surfacing data | 0.2 (NA) | 1.7 (100%) | 2.5 (65%) | 0.6 (23%) | 0.0 (NA) |
| Percentage behavioral coverage | 96.0 | 100.0 | 65.3 | 22.9 | 0.0 |
| **MdTag021** |  |  |  |  |  |
| Duration overall (days) | 0.2 | 1.7 | 3.8 | 2.4 | 5.2 |
| Days dive/surfacing data | 0.2 (NA) | 1.7 (100%) | 3.8 (100%) | 1.0 (40%) | 0.5 (NA) |
| Percentage behavioral coverage | 95.7 | 100.0 | 99.7 | 40.4 | 6.9 |

*Behavior data coverage for the days of dive/surfacing data was calculated by summing the total duration of all dive and surfacing periods for each phase and calculating the percentage of those durations out of the total duration of each phase. These are reported in parentheses as percentages on the Days dive/surfacing data lines. These percentages are not shown for Before and After as these are dependent on the start and end times of the deployment for each tag. The percentage of behavioral coverage is defined as the proportion of the duration of behavioral data relative to the duration of the tag within each phase.*

For the diel analysis of the dive data across SCC phases, day and night dive metrics were available for only Phase A and the interphase for both tags. Day dive rates decreased between Phase A and the interphase for both tags, and the percentage of surface time during day hours concurrently increased (Table S3, Figure S1). Night dive rates also decreased between Phase A and the interphase for both tags, and the percentage of surface time during night hours increased by roughly the same degree that it did during the day hours. Dive depths and durations did not vary significantly between phases for either the day or night.

Table S3 - A comparison of daytime and nighttime diving parameters from Blainville’s beaked whales exposed to MFAS for phases that meet the required coverage cutoff (Phase A and Interphase only).

| **Dive parameter per individual** | **Phase A** | **Interphase** | **Kruskal-Wallis Test p-value*** | **Post-hoc Dunn’s test significant pairs** |
| --- | --- | --- | --- | --- |
| ***Day*** *dive rate (dives/hour)* | | | | |
| MdTag020 | 1.9 | 1.5 | - |  |
| MdTag021 | 1.94 | 1.59 | - |  |
| ***Night*** *dive rate (dives/hour)* | | | | |
| MdTag020 | 1.43 | 1.11 | - |  |
| MdTag021 | 1.34 | 1.13 | - |  |
| *% time in surface periods at* ***day*** | | | | |
| MdTag020 | 38.02 | 45.83 | - |  |
| MdTag021 | 35.5 | 47.75 | - |  |
| *% time in surface periods at* ***night*** | | | | |
| MdTag020 | 52.74 | 59.62 | - |  |
| MdTag021 | 53.78 | 58.11 | - |  |
| *Median dive depth* ***day*** *(m)* | | | | |
| MdTag020 | 149.5 | 131.5 | 0.9669 | NA |
| MdTag021 | 127.5 | 117.5 | 0.5205 | NA |
| *Median dive depth* ***night*** *(m)* | | | | |
| MdTag020 | 135.5 | 111.5 | 0.1426 | NA |
| MdTag021 | 123.5 | 107.5 | 0.1063 | NA |
| *Median dive duration* ***day*** *(min)* | | | | |
| MdTag020 | 12.1 | 11.22 | 0.5223 | NA |
| MdTag021 | 12.23 | 10.85 | 0.0986 | NA |
| *Median dive duration* ***night*** *(min)* | | | | |
| MdTag020 | 12.87 | 11.08 | 0.5801 | NA |
| MdTag021 | 11.57 | 12 | 0.9958 | NA |

*None of the statistical tests were significant comparing dive depths or durations across SCC Phases.*


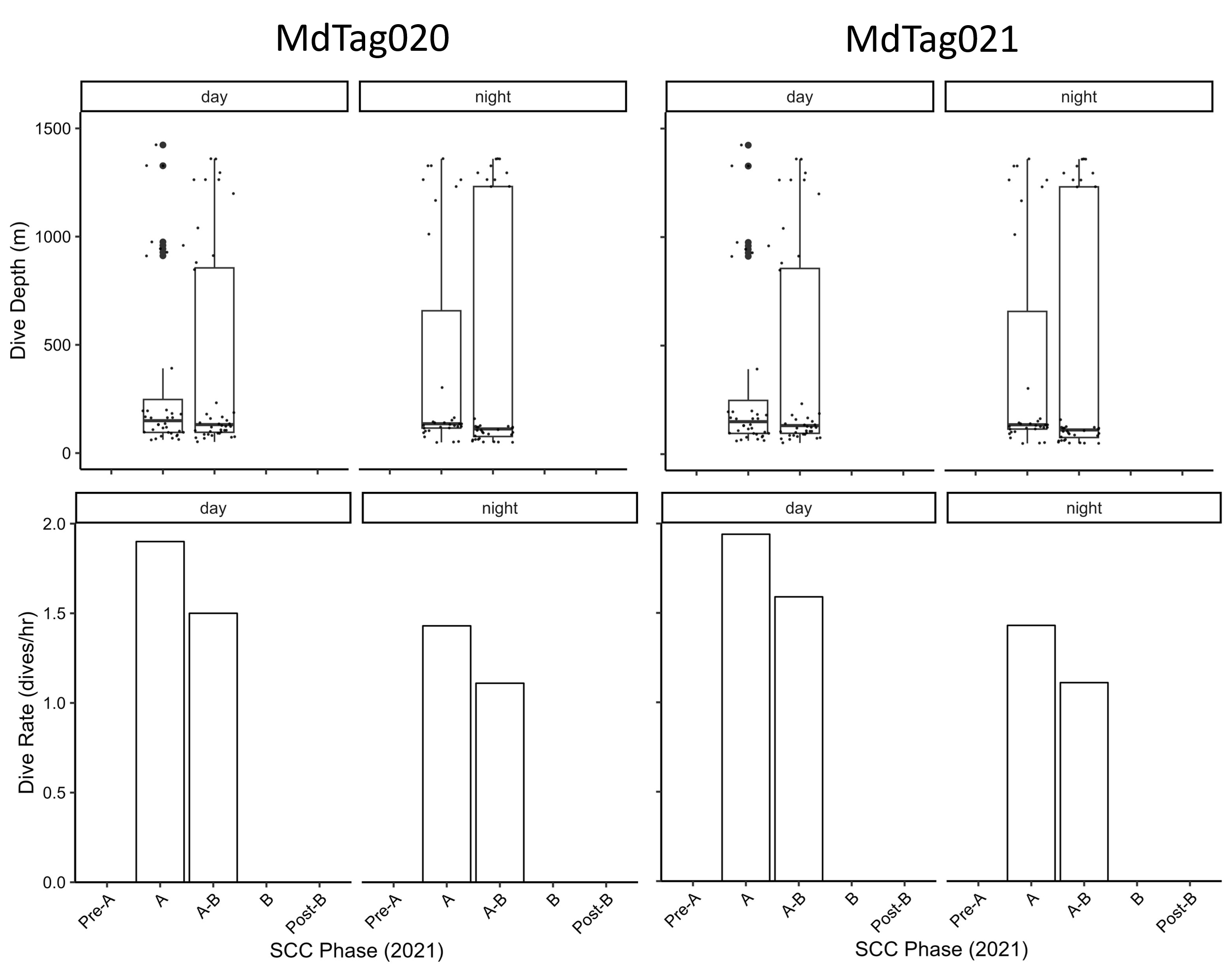


Figure S4 – Top Left. Boxplot showing dive depths of MdTag020 by SCC Phase and time of day. Bottom Left. Barplot showing dive rates of MdTag020 by SCC Phase and time of day. Top RIght. Boxplot showing dive depths of MdTag021 by SCC Phase and time of day. Bottom Right. Bagplot showing dive rates of MdTag021 by SCC Phase and time of day. Phase A-B refers to the Interphase.

# Kruskal-Wallis test results for the full tracks

Table S4- Chi-square (top) and p-values (bottom) from the Kruskal-Wallis tests that examined track movement variables in the SCC Phases Before, Phase A, Interphase, Phase B, and After for the full tracks. Significant p-values are in bold.

|  | MdTag020 | MdTag021 | MdTag022 |
| --- | --- | --- | --- |
| Bearing (deg) | 43.9  **<0.001** | 62.8  **<0.001** | 82.1  **<0.001** |
| Step  Length (m) | 22.7  **<0.001** | 13.7  0.009 | 6.6  0.08 |
| Speed (m/s) | 18.2  **0.001** | 36.7  **<0.001** | 42.8  **<0.001** |
| Turning Angle (rad) | 1.5  0.83 | 7.5  0.11 | 4.0  0.27 |
