# Supplementary material for: Exposure and response of satellite-tagged Blainville’s beaked whales to mid-frequency active sonar off Kaua‘i, Hawai‘i
Source: Mov Ecol. 2025 Apr 21;13:29. doi: 10.1186/s40462-025-00550-9 (PMC12010625; doi:10.1186/s40462-025-00550-9)
Supplement: Supplementary file 2 — Supplementary Materials 2. Hawaiian abstract. [file 40462_2025_550_MOESM2_ESM.docx]

# Abstract (Hawaiian Translation)

# Hōʻuluʻulu Manaʻo

## Ke Kahua

He pilikia a puni ka honua ka hāpane ʻana o nā koholā nuku i ke ana pīnaʻi hawewe kani a ka ʻOihana Moku ma muli hoʻi o nā hana pae wale i ka wā hoʻokahi o nā hanana hoʻomaʻamaʻa. ʻO ka maʻamau, lepili ʻia nā māmala kai loa me nā lepili miomio kūmanawa ma nā hoʻokolohua pā kāohi, a pā hoʻokahi manawa lākou i kahi pīnaʻi hawewe kani alapine waena (MFAS) pōkole. Ma kekahi ʻaoʻao, ma o ka loaʻa o nā lepili hoʻoili ukali kūloa a puni nā wahi ʻOihana Moku, hiki ke kālailai ʻia ka hana o nā holoholona i pā i nā hanana hoʻomaʻamaʻa maoli a ka ʻOihana Moku ma nā wā lōʻihi a ma nā henua pālākiō, me ka pā mau ʻana i nā kumu ʻokoʻa.

## Ke Kiʻina Hana

I mea e kālailai ai i ka lākou lawena i ka pā ʻana i nā wā hoʻolōʻihi ʻia o nā hanana hoʻomaʻamaʻa maoli a ka ʻOihana Moku, lepili ʻia aku nā lepili hoʻoili ukali ma ʻehā koholā nuku paʻapū (*Mesoplodon densirostris*) ma ka ponoō kao lele Pākīpika (PMRF) ma kai o Kauaʻi. Lepili ʻia nā lepili ma ʻekolu makahiki, mua o nā papa kauoha mokuluʻu (SCCs) me kekahi mau kumu MFAS. Hoʻohālike ʻia ka lawena luʻu o ʻelua mea i lepili pū ʻia me nā wā leo pūalu (GVPs) i lohe ʻia ma ia wahi. Hoʻohālike ʻia ka lawena luʻu ma mua o ka pā ʻana me ka lawena o nā wā e pā ana. Kālailai ʻia ka lawena holo papamoe ma ka hoʻohana ʻana i ka hōʻike mahuʻi ʻole Kruskal-Wallis a me ka hōʻike hoʻohālike nui Tukey-Kramer.

## Nā Hopena

Ua noho pū ʻelua koholā a lōkahi nō lāua i ka lawena luʻu a holo a hiki i ka wā MFAS, a ma ia wā nō i kaʻawale ai lāua. Hoʻopili ʻia he iwakāluakūmākolu luʻu ʻai hohonu i nā GVPs, e pēlā pū nō ʻekolu luʻu ʻana ma ka wā MFAS. Ma ka nānā ʻana i nā ʻikepili lawena luʻu, ʻo ka hohonu o hoʻokahi wale nō luʻu waena ka mea i noho ma waho aku o ke kanaiwakūmālima pākēneka lawena kahua ʻikepili. ʻEkolu mai loko mai o ka ʻehā ʻikepili lawena holo (75%) i ʻokoʻa mai ke kahua ʻikepili no hoʻokahi koholā ma nā wā SCCs, ʻokoʻa naʻe ka hāpane ma ka pākahi. Ma nā SCCs naʻe, ua noho nā koholā i loko o nā kilomika he ʻumi o ka PMRF, kokoke i nā wahi i hoʻohana ʻia ma mua a ma hope o nā SCCs.

## Nā Manaʻo Pani

Hōʻike kēia mau ʻikepili i kekahi mau loli hikiāpoko o ka lawena luʻu a me ka holo papamoe ma ka hāpane i nā MFAS. ʻAʻole naʻe i ʻike ʻia nā hāpane hōʻalo o kēia mau koholā nuku, eia kā noho lākou i kahi komohana o ia wahi ma ka wā MFAS a ua hoʻi kekahi i ia wahi ʻelua manawa ma hope o ka SCC. Koʻikoʻi nō ka lēpili ʻana a me ke kālailai kiʻi no ka maopopo ʻana i ka hana a noho ʻana o ke koholā nuku paʻapū i kona kaianoho a no ke ana ʻana i ka ke ʻano o ka hopena i ka pā mau ʻana i ka MFAS.
